# Partitioning the Heritability of Tourette Syndrome and Obsessive Compulsive Disorder Reveals Differences in Genetic Architecture

**DOI:** 10.1371/journal.pgen.1003864

**Published:** 2013-10-24

**Authors:** Lea K. Davis, Dongmei Yu, Clare L. Keenan, Eric R. Gamazon, Anuar I. Konkashbaev, Eske M. Derks, Benjamin M. Neale, Jian Yang, S. Hong Lee, Patrick Evans, Cathy L. Barr, Laura Bellodi, Fortu Benarroch, Gabriel Bedoya Berrio, Oscar J. Bienvenu, Michael H. Bloch, Rianne M. Blom, Ruth D. Bruun, Cathy L. Budman, Beatriz Camarena, Desmond Campbell, Carolina Cappi, Julio C. Cardona Silgado, Danielle C. Cath, Maria C. Cavallini, Denise A. Chavira, Sylvain Chouinard, David V. Conti, Edwin H. Cook, Vladimir Coric, Bernadette A. Cullen, Dieter Deforce, Richard Delorme, Yves Dion, Christopher K. Edlund, Karin Egberts, Peter Falkai, Thomas V. Fernandez, Patience J. Gallagher, Helena Garrido, Daniel Geller, Simon L. Girard, Hans J. Grabe, Marco A. Grados, Benjamin D. Greenberg, Varda Gross-Tsur, Stephen Haddad, Gary A. Heiman, Sian M. J. Hemmings, Ana G. Hounie, Cornelia Illmann, Joseph Jankovic, Michael A. Jenike, James L. Kennedy, Robert A. King, Barbara Kremeyer, Roger Kurlan, Nuria Lanzagorta, Marion Leboyer, James F. Leckman, Leonhard Lennertz, Chunyu Liu, Christine Lochner, Thomas L. Lowe, Fabio Macciardi, James T. McCracken, Lauren M. McGrath, Sandra C. Mesa Restrepo, Rainald Moessner, Jubel Morgan, Heike Muller, Dennis L. Murphy, Allan L. Naarden, William Cornejo Ochoa, Roel A. Ophoff, Lisa Osiecki, Andrew J. Pakstis, Michele T. Pato, Carlos N. Pato, John Piacentini, Christopher Pittenger, Yehuda Pollak, Scott L. Rauch, Tobias J. Renner, Victor I. Reus, Margaret A. Richter, Mark A. Riddle, Mary M. Robertson, Roxana Romero, Maria C. Rosàrio, David Rosenberg, Guy A. Rouleau, Stephan Ruhrmann, Andres Ruiz-Linares, Aline S. Sampaio, Jack Samuels, Paul Sandor, Brooke Sheppard, Harvey S. Singer, Jan H. Smit, Dan J. Stein, E. Strengman, Jay A. Tischfield, Ana V. Valencia Duarte, Homero Vallada, Filip Van Nieuwerburgh, Jeremy Veenstra-VanderWeele, Susanne Walitza, Ying Wang, Jens R. Wendland, Herman G. M. Westenberg, Yin Yao Shugart, Euripedes C. Miguel, William McMahon, Michael Wagner, Humberto Nicolini, Danielle Posthuma, Gregory L. Hanna, Peter Heutink, Damiaan Denys, Paul D. Arnold, Ben A. Oostra, Gerald Nestadt, Nelson B. Freimer, David L. Pauls, Naomi R. Wray, S. Evelyn Stewart, Carol A. Mathews, James A. Knowles, Nancy J. Cox, Jeremiah M. Scharf

**Affiliations:** 1 Section of Genetic Medicine, Department of Medicine, University of Chicago, Chicago, Illinois, United States of America; 2 Psychiatric and Neurodevelopmental Genetics Unit, Center for Human Genetics Research, Department of Psychiatry, Harvard Medical School, Massachusetts General Hospital, Boston, Massachusetts, United States of America; 3 Stanley Center for Psychiatric Research, Broad Institute of Harvard and MIT, Cambridge, Massachusetts, United States of America; 4 Department of Medicine, University of Chicago, Chicago, Illinois, United States of America; 5 Department of Human Genetics, University of Chicago, Chicago, Illinois, United States of America; 6 Department of Psychiatry, Academic Medical Center, University of Amsterdam, Amsterdam, The Netherlands; 7 Analytic and Translational Genetics Unit, Massachusetts General Hospital, Boston, Massachusetts, United States of America; 8 The University of Queensland, Diamantina Institute, Queensland, Australia; 9 The University of Queensland, Queensland Brain Institute, Queensland, Australia; 10 The Toronto Western Research Institute, University Health Network, Toronto, Ontario, Canada; 11 The Hospital for Sick Children, Toronto, Ontario, Canada; 12 Università Vita-Salute San Raffaele, Milano, Italy; 13 Herman Dana Division of Child and Adolescent Psychiatry, Hadassah-Hebrew University Medical Center, Jerusalem, Israel; 14 Universidad de Antioquia, Universidad Pontificia Bolivariana, Medellín, Colombia; 15 Department of Psychiatry and Behavioral Sciences, Johns Hopkins University School of Medicine, Baltimore, Maryland, United States of America; 16 Department of Psychiatry, Yale University, New Haven, Connecticut, United States of America; 17 Child Study Center, Yale University School of Medicine, New Haven, Connecticut, United States of America; 18 North Shore-Long Island Jewish Medical Center, Manhasset, New York, United States of America; 19 New York University Medical Center, New York, New York, United States of America; 20 North Shore-Long Island Jewish Health System, Manhasset, New York, United States of America; 21 Hofstra University School of Medicine, Hempstead, New York, United States of America; 22 Instituto Nacional de Psiquiatría Ramon de la Fuente Muñiz, Mexico City, Mexico; 23 University College London, London, United Kingdom; 24 Department of Psychiatry, University of Hong Kong, Hong Kong, China; 25 Department of Psychiatry, University of São Paulo Medical School, São Paulo, Brazil; 26 Department of Psychiatry, VU University Medical Center, Amsterdam, The Netherlands; 27 Department of Clinical & Health Psychology, Utrecht University, Utrecht, The Netherlands; 28 Altrecht Academic Anxiety Center, Utrecht, The Netherlands; 29 Ospedale San Raffaele, Milano, Italy; 30 Department of Psychology, University of California Los Angeles, Los Angeles, California, United States of America; 31 Department of Psychiatry, University of California San Diego, La Jolla, California, United States of America; 32 University of Montreal, Montreal, Quebec, Canada; 33 Department of Preventative Medicine, Division of Biostatistics, Keck School of Medicine, University of Southern California, Los Angeles, California, United States of America; 34 Institute for Juvenile Research, Department of Psychiatry, University of Illinois at Chicago, Chicago, Illinois, United States of America; 35 Laboratory of Pharmaceutical Biotechnology, Ghent University, Ghent, Belgium; 36 Human Genetics and Cognitive Functions, Institut Pasteur, Paris, France; 37 Fondation Fondamental, French National Science Foundation, Creteil, France; 38 AP-HP, Robert Debré Hospital, Department of Child and Adolescent Psychiatry, Paris, France; 39 Department of Psychiatry, University of Montreal, Montreal, Quebec, Canada; 40 Department of Child and Adolescent Psychiatry, Psychosomatics and Psychotherapy, University of Würzburg, Würzburg, Germany; 41 Department of Psychiatry and Psychotherapy, University of Munich, Munich, Germany; 42 Department of Psychiatry, Yale University School of Medicine, New Haven, Connecticut, United States of America; 43 Clinica Herrera Amighetti, Avenida Escazú, San José, Costa Rica; 44 OCD Program, Department of Psychiatry, Massachusetts General Hospital, Harvard Medical School, Boston, Massachusetts, United States of America; 45 Department of Psychiatry and Psychotherapy, Helios-Hospital Stralsund, University Medicine Greifswald, Greifswald, Germany; 46 Department of Psychiatry and Human Behavior, Brown Medical School, Butler Hospital, Providence, Rhode Island, United States of America; 47 Neuropediatric Unit, Shaare Zedek Medical Center, Jerusalem, Israel; 48 Department of Genetics, Human Genetics Institute of New Jersey, Rutgers University, Piscataway, New Jersey, United States of America; 49 Department of Psychiatry, University of Stellenbosch, Stellenbosch, South Africa; 50 Department of Psychiatry, Faculdade de Medicina da Universidade de Säo Paulo, Brazil; 51 Parkinson's Disease Center and Movement Disorders Clinic, Department of Neurology, Baylor College of Medicine, Houston, Texas, United States of America; 52 Department of Psychiatry, Massachusetts General Hospital, Boston, Massachusetts, United States of America; 53 Neurogenetics Section, Centre for Addiction and Mental Health, Toronto, Ontario, Canada; 54 Department of Psychiatry, University of Toronto, Toronto, Ontario, Canada; 55 Yale Child Study Center, Department of Genetics, Yale University School of Medicine, New Haven, Connecticut, United States of America; 56 Atlantic Neuroscience Institute, Overlook Hospital, Summit, New Jersey, United States of America; 57 Carracci Medical Group, Mexico City, Mexico; 58 Institut Mondor de Recherche Biomédicale, Psychiatric Genetics, Créteil, France; 59 Child Study Center, Psychiatry, Pediatrics and Psychology, Yale University, New Haven, Connecticut, United States of America; 60 Department of Psychiatry and Psychotherapy, University of Bonn, Bonn, Germany; 61 Department of Psychiatry, Institute of Human Genetics, University of Illinois at Chicago, Chicago, Illinois, United States of America; 62 MRC Unit on Anxiety & Stress Disorders, Department of Psychiatry, University of Stellenbosch, Stellenbosch, South Africa; 63 Department of Psychiatry, University of California at San Francisco, San Francisco, California, United States of America; 64 Department of Psychiatry and Human Behavior, School of Medicine, University of California Irvine (UCI), Irvine, California, United States of America; 65 University of Utah, Salt Lake City, Utah, United States of America; 66 Laboratory of Clinical Science, NIMH Intramural Research Program, Bethesda, Maryland, United States of America; 67 Department of Clinical Research, Medical City Dallas Hospital, Dallas, Texas, United States of America; 68 Department of Psychiatry, Rudolf Magnus Institute of Neuroscience, University Medical Center, Utrecht, The Netherlands; 69 Center for Neurobehavioral Genetics, Semel Institute for Neuroscience and Human Behavior, University of California Los Angeles, Los Angeles, California, United States of America; 70 Department of Genetics, Yale University School of Medicine, New Haven, Connecticut, United States of America; 71 Department of Psychiatry and the Behavioral Sciences, Zilkha Neurogenetic Institute, Keck School of Medicine, University of Southern California, Los Angeles, California, United States of America; 72 Department of Psychiatry and Biobehavioral Sciences, University of California, Los Angeles, David Geffen School of Medicine, Los Angeles, California, United States of America; 73 Departments of Psychiatry and Psychology and the Child Study Center, Yale University, New Haven, Connecticut, United States of America; 74 Partners Psychiatry and McLean Hospital, Boston, Massachusetts, United States of America; 75 Frederick W. Thompson Anxiety Disorders Centre, Sunnybrook Health Sciences Centre, Toronto, Ontario, Canada; 76 St George's Hospital and Medical School, London, United Kingdom; 77 Hospital Nacional de Niños, San Jose, Costa Rica; 78 Child and Adolescent Psychiatry Unit (UPIA), Department of Psychiatry, Federal University of São Paulo, São Paulo, Brazil; 79 Department of Psychiatry & Behavioral Neurosciences, Wayne State University and the Detroit Medical Center, Detroit, Michigan, United States of America; 80 Montreal Neurological Institute, McGill University, Montreal, Quebec, Canada; 81 Department of Psychiatry and Psychotherapy, University of Cologne, Cologne, Germany; 82 University Health Care Services - SMURB, Universidade Federal da Bahia, Salvador, Bahia, Brazil; 83 Department of Psychiatry, University of Toronto and University Health Network, Toronto Western Research Institute and Youthdale Treatment Centers, Toronto, Ontario, Canada; 84 Johns Hopkins University School of Medicine, Baltimore, Maryland, United States of America; 85 University of Cape Town, Cape Town, South Africa; 86 Department of Medical Genetics, University Medical Center Utrecht, Utrecht, The Netherlands; 87 Departments of Psychiatry, Pediatrics, and Pharmacology, Kennedy Center for Research on Human Development, and Brain Institute, Vanderbilt University, Nashville, Tennessee, United States of America; 88 Department of Child and Adolescent Psychiatry, University of Zurich, Zurich, Switzerland; 89 Department of Child and Adolescent Psychiatry, University of Würzburg, Würzburg, Germany; 90 Department of Psychiatry, Academic Medical Center and Netherlands Institute for Neuroscience, an Institute of the Royal Netherlands Academy of Arts and Sciences (NIN-KNAW), Amsterdam, The Netherlands; 91 Unit on Statistical Genomics, NIMH Intramural Research Program, Bethesda, Maryland, United States of America; 92 Department of Psychiatry, University of Utah, Salt Lake City, Utah, United States of America; 93 National Institute of Genomic Medicine-SAP, Carracci Medical Group, Mexico City, Mexico; 94 Department of Functional Genomics, Center for Neurogenomics and Cognitive Research, Neuroscience Campus Amsterdam, VU University Amsterdam, De Boelelaan, Amsterdam, The Netherlands; 95 Department of Clinical Genetics, VU Medical Centre, De Boelelaan, Amsterdam, The Netherlands; 96 Department of Child and Adolescent Psychiatry, Erasmus University Medical Centre, Rotterdam, The Netherlands; 97 Department of Psychiatry, University of Michigan, Ann Arbor, Michigan, United States of America; 98 Section of Medical Genomics, Department of Clinical Genetics, VU University Medical Center Amsterdam, The Netherlands; 99 German Center for Neurodegenerative Diseases, Tübingen, Germany; 100 Netherlands Institute for Neuroscience, an Institute of the Royal Netherlands Academy of Arts and Sciences (NIN-KNAW), Amsterdam, The Netherlands; 101 Program in Genetics and Genome Biology, The Hospital for Sick Children, Toronto, Ontario, Canada; 102 Department of Clinical Genetics, Erasmus Medical Center, Rotterdam, The Netherlands; 103 British Columbia Mental Health and Addictions Research Institute, University of British Columbia, Vancouver, British Columbia, Canada; 104 Division of Cognitive and Behavioral Neurology, Brigham and Womens Hospital, Boston, Massachusetts, United States of America; 105 Department of Neurology, Massachusetts General Hospital, Boston, Massachusetts, United States of America; University of Colorado Boulder, United States of America

## Abstract

The direct estimation of heritability from genome-wide common variant data as implemented in the program Genome-wide Complex Trait Analysis (GCTA) has provided a means to quantify heritability attributable to all interrogated variants. We have quantified the variance in liability to disease explained by all SNPs for two phenotypically-related neurobehavioral disorders, obsessive-compulsive disorder (OCD) and Tourette Syndrome (TS), using GCTA. Our analysis yielded a heritability point estimate of 0.58 (se = 0.09, p = 5.64e-12) for TS, and 0.37 (se = 0.07, p = 1.5e-07) for OCD. In addition, we conducted multiple genomic partitioning analyses to identify genomic elements that concentrate this heritability. We examined genomic architectures of TS and OCD by chromosome, MAF bin, and functional annotations. In addition, we assessed heritability for early onset and adult onset OCD. Among other notable results, we found that SNPs with a minor allele frequency of less than 5% accounted for 21% of the TS heritability and 0% of the OCD heritability. Additionally, we identified a significant contribution to TS and OCD heritability by variants significantly associated with gene expression in two regions of the brain (parietal cortex and cerebellum) for which we had available expression quantitative trait loci (eQTLs). Finally we analyzed the genetic correlation between TS and OCD, revealing a genetic correlation of 0.41 (se = 0.15, p = 0.002). These results are very close to previous heritability estimates for TS and OCD based on twin and family studies, suggesting that very little, if any, heritability is truly missing (i.e., unassayed) from TS and OCD GWAS studies of common variation. The results also indicate that there is some genetic overlap between these two phenotypically-related neuropsychiatric disorders, but suggest that the two disorders have distinct genetic architectures.

## Author Summary

Family and twin studies have shown that genetic risk factors are important in the development of Tourette Syndrome (TS) and obsessive compulsive disorder (OCD). However, efforts to identify the individual genetic risk factors involved in these two neuropsychiatric disorders have been largely unsuccessful. One possible explanation for this is that many genetic variations scattered throughout the genome each contribute a small amount to the overall risk. For TS and OCD, the genetic architecture (characterized by the number, frequency, and distribution of genetic risk factors) is presently unknown. This study examined the genetic architecture of TS and OCD in a variety of ways. We found that rare genetic changes account for more genetic risk in TS than in OCD; certain chromosomes contribute to OCD risk more than others; and variants that influence the level of genes expressed in two regions of the brain can account for a significant amount of risk for both TS and OCD. Results from this study might help in determining where, and what kind of variants are individual risk factors for TS and OCD and where they might be located in the human genome.

## Introduction

For most complex traits, DNA sequence variants that meet the genome-wide significance threshold do not explain the majority of the heritability as estimated by twin and family studies [1]. Heritability (broad sense) is defined as the proportion of phenotypic variance accounted for by genotypic variance within a population. Narrow sense heritability is a special case of broad sense heritability and refers to the proportion of phenotypic variance that is due only to *additive* genetic effects. The limited heritability explained by significant GWAS findings has led to the so-called “missing heritability” dilemma and subsequent hypotheses have been generated for how to capture the heritable factors contributing to human trait variation [2], [3]. However, others have argued that the proportion of heritability explained by “top GWAS hits” is limited by currently available sample sizes and analytic approaches, and that sub-threshold GWAS signals may capture a much larger proportion of heritability [1], [4]. Indeed, under current experimental conditions, genome-wide significant GWAS findings alone are likely to account for a very small proportion of total risk variants for many complex disorders and by extension a small proportion of heritability.

The application of genome-wide estimation of heritability using restricted maximum likelihood (REML) methods has provided a new means to quantify narrow sense heritability attributable to all interrogated variants in GWAS [5]. This approach, as implemented in the Genome-wide Complex Trait Analysis (GCTA) package, has been utilized to study a number of complex human phenotypes including autism, schizophrenia, height, Parkinson's disease, type 2 diabetes, and hypertension, and has shown that a significant proportion of genetic risk undiscovered by GWAS was nevertheless detectable by REML heritability approaches [5], [6], [7], [8].

Tourette Syndrome (TS) and obsessive-compulsive disorder (OCD) are neurodevelopmental disorders with overlapping neural circuitries and similarities in phenotypic expression [9], [10], [11]. Neuroimaging studies have implicated specific brain regions, i.e. the ventromedial prefrontal cortex (VMPFC), anterior cingulate cortex (ACC), orbitofrontal cortex (OFC), parietal cortex and somatosensory cortex, along with the striatum and the thalamus, as being involved in the pathophysiology of both OCD and TS [12]. These brain regions are interconnected in multiple recurrent loops, making up the cortico-striatal-thalamo-cortical (CSTC) circuitry, and are thought to be involved in action selection, performance monitoring, response inhibition, and goal-directed behaviors [13], [14]. Both TS and OCD have a strong familial component, and often co-occur within families. Multiple studies have suggested that OCD and TS are both highly heritable (h^2^ = 27%–45% adult onset OCD; 65% for childhood onset OCD, h^2^ = 60% for TS) and likely to be genetically related [15]–[18], [19], [20], [21], [22], [23]–[25], [26], [27], [28], [29]. For review of TS heritability studies see Scharf and Pauls, 2007.

This study sought to quantify the heritability of both TS and OCD using genome wide genotype data and the REML approach implemented in GCTA [5]. Here we present results from a comprehensive heritability study of these disorders using thorough and stringent quality controls. In addition to obtaining a direct genetic estimate of total heritability for each trait, we also examined the genetic architectures of TS and OCD by partitioning genetic variation according to minor allele frequency, chromosome, and functional annotation. Functional annotations included annotation by genic regions as well as annotation of SNPs correlated with gene expression in parietal cortex and cerebellum, two brain regions for which we had previously generated eQTL data. In addition, we assessed heritability for early onset and adult onset OCD. Lastly, we conducted a bivariate analysis to examine the genetic correlation between OCD and TS.

## Methods

### Ethics Statement

All participants 18 years of age and older gave informed consent. Individuals under 18 years of age gave assent after a parent signed a consent form on their behalf. The Ethics Committees of each participating site approved this research and the research was conducted in accordance with the Declaration of Helsinki.

### Sample

The datasets used in this study are described in depth elsewhere [30], [31]. Briefly, DNA from individuals with TS or OCD and from controls was randomized across plates and genotyped using the Illumina Human610-Quad genotyping array. Additional unscreened controls that were genotyped as a part of the SAGE (genotyped on Illumina HumanHap1Mv1_C) and iControl (genotyped on Illumina HumanHap550v1/v3) datasets were also included in this study. To reduce effects of population stratification, subjects were limited to those with genetically defined European ancestry, based on principal components clustering analysis using genome-wide pairwise identity-by-descent (IBD) information as estimated with EIGENSTRAT 3.0 [32] and including previously defined European population samples as reference (HapMap3.0).

### Quality Control

The first phase of quality control analyses, including assessment of Hardy-Weinberg equilibrium, differential missingness, platform effects, population stratification, and genotyping call rate, was conducted as a part of the recently published GWAS of OCD and TS [30], [31]. The variance components models in the REML analysis utilized all unpruned genotype data simultaneously. Because all genotypes are fitted together in a given variance component, these components are particularly susceptible to minor technical and experimental artifacts that might only modestly affect each genotype (i.e., in a SNP-by-SNP test of association) but could have a substantial cumulative global effect on the results from a mixed linear model. We thus undertook additional, more stringent quality control measures to minimize any possible persistent population stratification and experimental bias. Prior to case-control comparisons, we first focused solely on the control dataset to develop our QC pipeline. We split the controls by data source (iControl vs. SAGE controls) and performed the following QC steps using PLINK. We implemented stringent thresholds and removed additional SNPs showing low levels of differential missingness between cases and controls (p<0.05), modest deviation from Hardy-Weinberg expectation (p<0.05), and significant platform effect after adjustment for all ten principal components (p<0.001). In addition, individuals with genotype call rate <99.9%, or with a high degree of relatedness (pi-hat>0.05) were removed (Table S1). To assess any residual cross-platform artifacts that might artificially elevate the heritability estimate, we conducted a dummy case-control GWAS by assigning case status to the iControl data (N = 1,104) and control status to the SAGE Controls (N = 2,190). We detected no significant association with platform “phenotype” by logistic regression **(Figure S1)** or “heritability” between cross-platform controls (h^2^ = 10^−6^, se = 0.11) (Table S2). Additionally, we analyzed ten permutations of the dummy case phenotype and detected no significant heritability in any permuted analysis. In addition to these QC steps, we examined the data for any possible residual population stratification or cryptic relatedness, which is described in depth in the Supplementary Methods **(Figures S1, S2, S3, S4)**. The quality control and matching steps resulted in a final data set of 617 TS cases and 4,116 TS controls genotyped on 393,387 SNPs, as well as 1,061 OCD cases and 4,236 OCD controls genotyped on 373,846 SNPs. Each analysis included the top 20 principal components as covariates.

### Heritability Analysis

For each analysis presented, GCTA v1.2 ([5]; www.complextraitgenomics.com) was used to create a genetic relationship matrix (GRM) file containing IBD relationship calculations for all pair-wise sets of individuals. Principal components were determined within GCTA, using all genotype data, and the top 20 principal components were applied to each analysis. The REML analysis was then performed using the respective GRMs and principal component quantitative covariates. As this analysis was performed with dichotomous case/control traits, it was necessary to convert the phenotypic variance to an underlying liability scale. This conversion uses population prevalence to adjust for case/control ascertainment in the sample and to modify the phenotypic variance estimate accordingly [4]. We conducted primary analyses using 2.5% for OCD prevalence and 0.8% for TS. As a range of prevalence estimates for both OCD and TS are frequently reported, we conducted additional sensitivity analyses to examine the heritability estimates for TS and OCD across a range of reported prevalences **(Table S3)** [33], [34], [35], [36]. Additionally, we provide heritability results converted to the sibling relative risk scale for further interpretation **(Table S4)**. We conducted three primary analyses (univariate TS heritability, univariate OCD heritability, joint OCD and TS bivariate analysis) and five exploratory analyses (partitioning by chromosome, MAF, genic annotation, brain eQTL annotation, age of onset). For each primary analysis, ten permutations of the phenotype were performed and GCTA was run on each permutation to observe the stability of the heritability estimate.

### Bivariate OCD and TS Analysis

In addition, we calculated the genetic correlation between OCD and TS using the GCTA bivariate REML analysis. We split the shared control sample between the TS cases and the OCD cases in a manner that preserved the matched ancestry structure and the proportion of cases to controls for each disorder. An initial analysis included co-morbid TS and OCD cases assigned to either the TS or OCD samples based on their primary diagnosis as determined by the clinical team. We conducted a secondary bivariate analysis limiting the SNPs included to a subset of SNPs previously identified as regulators of gene expression in the brain. A final sensitivity analysis was conducted after removing all 316 case samples with known overlapping comorbidity (83 OCD samples with TS or chronic tics, and 233 TS samples with OCD) to assess the effect of co-morbidity on the cross-disorder genetic correlation. We then applied a likelihood ratio test (LRT) to determine the statistical significance of each genetic correlation.

### Imputation Analysis

Imputation was performed using IMPUTE v2.1.2 and the 1000 Genomes Project data as a reference panel. Only imputed SNPs that were in strong linkage disequilibrium (LD) (info>0.6) with genotyped SNPs and had a high certainty (>90%) of the predicted genotypes were retained. Imputed SNPs that showed significant genotyping platform effects were excluded. Imputed results were converted to MaCH format (i.e., .mldose, .mlinfo) using an in-house script. MaCH dosage data was used to create GRMs for each chromosome. Chromosome specific GRMs were then merged as needed for additional analyses. The total number of imputed SNPs after QC included 7,657,106 SNPs in both the TS and OCD samples.

### Partitioning Heritability

#### By chromosome

A separate GRM was generated for each chromosome. Each GRM was then run in separate REML analysis. An additional analysis was conducted in which all chromosomes were modeled jointly in a single REML analysis.

#### By minor allele frequency

We chose not to employ a minor allele frequency (MAF) cutoff in any of the heritability analyses. This decision was based on the observation that minor allele frequency cutoffs did not alter estimates of heritability for the control-control analysis after establishment of stringent differential missingness rates and call rate. We partitioned the directly genotyped and imputed variants according to MAF bin. For the directly genotyped variants we created six bins representing MAFs from 0.001–.05, >0.05–0.1, >.1–.2, >.2–.3, >.3–.4, and >.4–.5 and generated GRMs for each bin. For the imputed genotypes we created two bins representing MAF 0.001–0.05 and >0.05 to 0.5 and generated GRMs for each bin. For each set of variants (directly genotyped and imputed respectively) we then combined binned GRMs in a single joint REML analysis, allowing the effects of LD to be partitioned by the REML analytic approach.

#### By functional annotation

We annotated variants for genic and intergenic classification using ANNOVAR (hg18, refGene) [37]. Genic variants included all those variants annotated to exons, introns, UTRs and splice sites. Intergenic variants included those not otherwise annotated as genic. Additionally, we annotated directly genotyped and imputed SNPs that we had previously identified as significantly associated with gene expression (p<0.001) in parietal cortex, (GSE35977), cerebellum (GSE35974), and skeletal muscle (GSE40234). Details of the eQTL detection are described in supplementary methods and in previous publications [38]–[45]. Three sets of analyses were conducted using the eQTL annotations. The first analysis simply partitioned the parietal eQTLs and cerebellar eQTLs from their respective complements for all imputed SNPs. The second model included four partitions: 1) brain only eQTLs (those found in cerebellum or parietal tissues but not in muscle), 2) muscle only eQTLs (those found in muscle and not in either brain tissue), 3) eQTLs common to brain and muscle, and 4) a final partition with non-eQTL SNPs. The last analysis included four total partitions to accommodate eQTLs exclusive to each brain tissue (cerebellum and parietal) as well as eQTLs found in both brain tissues, and the remainder of all imputed SNPs. Annotations were applied to the TS and OCD case/control data and used to create partitions. This resulted in a total of four separate annotation-based REML analyses. For each analysis, we created a single GRM for each partition. Finally, for each analysis, we included the functional variant GRM(s) and the respective complement GRM together in one joint REML analysis.

#### Age of onset subset (OCD)

Multiple studies have reported significantly higher heritability for early-onset OCD than for adult onset OCD [16], [46]. Hanna and colleagues (2005) [47] suggested a possible threshold of 14 years to define early-onset OCD, however, as our data was collected retrospectively, potentially introducing a recall bias, we chose to employ a conservative threshold for early-onset of symptoms or diagnosis at age 16. We sought to test the hypothesis that early-onset OCD is more highly heritable than adult-onset OCD by dividing the OCD sample based on symptom onset or age at diagnosis (≤16 = early onset, >16 = adult-onset). A total of 732 cases were diagnosed or reported symptom onset prior to age 16 and were considered early onset. A total of 267 cases were diagnosed or exhibited symptoms later than age 16 and were classified as adult onset. Age of onset data was missing for 62 cases. GCTA analysis was performed on both subsets of samples.

## Results

### Univariate Heritability Analyses of TS and OCD

Analysis of the control datasets split by platform demonstrated no artifactual “cross-platform” heritability (h^2^ = 0.000001; se = 0.11, p = 0.5) **(Table S2)**. The overall narrow-sense heritability for TS calculated using the directly genotyped data of 617 TS cases and 4,116 controls was 0.58 (se = 0.09, p = 5.64e-12) and for OCD (1,061 cases, 4,236 controls) was 0.37(se = 0.07, p = 1.5e-07) **(** **Table 1** **)**. In order to test for possible inflation in the TS heritability point estimate due to small sample size, the OCD analysis was repeated using a random set of 617 OCD cases that matched the TS sample size. This experiment yielded a near-identical heritability point estimate for OCD with an expected increase in the standard error (h^2^ = 0.36; se = 0.12, p = 0.0009). For each primary analysis, ten permutations of the phenotype were conducted as an additional control, yielding on average no significant heritability (h^2^_TS_ = 0.06, se = 0.07, p = 0.3; h^2^_OCD_ = 0.06, se = 0.08, p = 0.3). Analyses were also conducted on imputed data, resulting in similar estimates of heritability for TS (0.48, se = 0.09, p = 3.0e-08) and OCD (0.32, se = 0.07, p = 7e-06).

**Table 1:** Overall heritability analysis of obsessive-compulsive disorder and Tourette syndrome.

Diagnosis	Number of Cases	Number of Controls	Total Number of Individuals	Number of SNPs	Heritability Estimate (*se*)	p-value
TS	617	4,116	4,733	393,387	0.58 (*0.09*)	5.64e-12
TS Imputation	617	4,116	4,733	7,782,687	0.48 (*0.09*)	3.0e-08
OCD	1,061	4,236	5,297	373,846	0.37 (*0.07*)	1.5e-07
Childhood Onset OCD (≤16 yrs old)	732	3,985	4,717	373,846	0.43 (*0.10*)	1e-05
Adult Onset (>16 yrs old)	267	4,200	4,467	373,846	0.26 (0.24)	0.1
OCD**	617	4,355	4,972	373,846	0.36 (*0.12*)	0.0009
OCD Imputation	1,061	4,236	5,297	7,850,541	0.32 (*0.07*)	7e-06
Control-Control	1,166	2,457	3,294	392,120	0.0000001 (*0.06*)	0.5
TS Permutations*	617	4,116	4,733	393,387	0.06 (*0.07*)	0.3
OCD Permutations*	1,061	4,236	5,297	373,846	0.06 (*0.08*)	0.3

Legend: se: standard error; SNPs: single nucleotide polymorphisms; TS: Tourette syndrome; OCD: Obsessive-compulsive disorder;

* Average of 10 analyses of permuted phenotypes.

** Sample size reduced to match size of TS sample.

### Genetic Correlation between TS and OCD

A bivariate analysis of the TS and OCD samples using directly genotyped data yielded similar estimates for the heritability of TS (0.51, se = 0.10) and OCD (0.43, se = 0.08). The genetic correlation between the two disorders was 0.41 (se = 0.15), which was significantly different from zero (LRT = 7.98; p = 0.002). We conducted an exploratory bivariate analysis which limited the included SNPs to eQTLs identified in parietal cortex or cerebellum and found a genetic correlation of 0.31 (se = 0.17) which was also significantly different from zero (LRT = 3.62, p = 0.03). Our assessment of the impact of overlapping phenotypic co-morbidity on the estimate of genetic correlation resulted in a smaller, yet purer set of samples (after removing 316 samples with known TS/tic and OCD co-morbidity) and yielded a genetic correlation of 0.50 (se = 0.29; LRT = 4.08; p = 0.02).

### Partitioned Analysis by Chromosome

For both the TS and OCD phenotypes, the summed total of individual “by chromosome” heritability estimates (h^2^_TS_ = 0.61, h^2^_OCD_ = 0.35) were not different than the global univariate heritability estimates (h^2^_TS_ = 0.58, h^2^_OCD_ = 0.37) **(Table S5 and S6)**. These results suggest that population stratification was appropriately controlled in these analyses.

**Figure 1:**
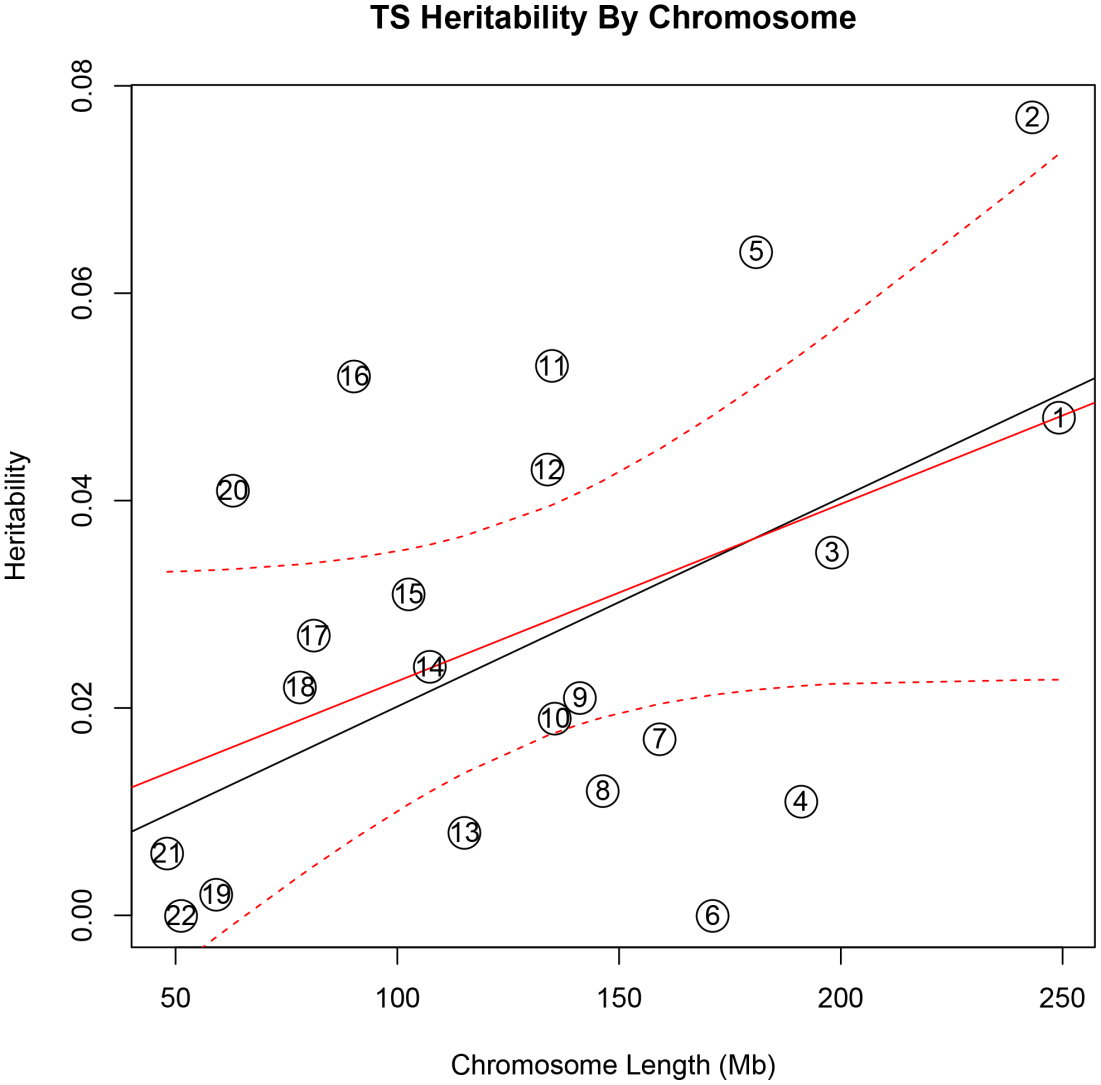
Tourette Syndrome heritability by chromosome. Heritability (y-axis) per chromosome is plotted against chromosome length (x-axis). The red line represents heritability regressed on chromosome length and the 95% confidence interval around the slope of the regression model is represented by the red dashed lines. The black line represents the expected heritability per chromosome (based on size) regressed on chromosome length. Chromosomes 2, 5, 11, 12, 16, and 20 fall outside of the 95% confidence interval and appear to account for more heritability than expected based on chromosome length.

**Figure 2:**
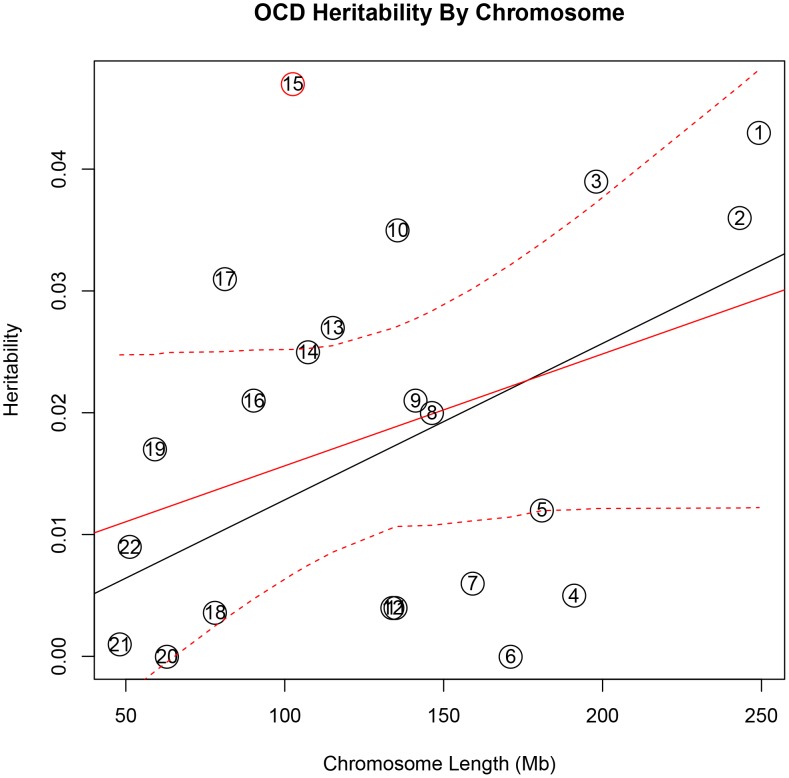
Obsessive-compulsive disorder heritability by chromosome. Heritability (y-axis) per chromosome is plotted against chromosome length (x-axis). The red line represents heritability regressed on chromosome length and the 95% confidence interval around the slope of the regression model is represented by the red dashed lines. The black line represents the expected heritability per chromosome (based on size) regressed on chromosome length. Chromosome 15 is shown in red to highlight its extreme deviation from the expected heritability based on chromosome length. Chromosomes 3, 10, 13, and 17 are also outside of the 95% interval and appear to account for more heritability than expected based on chromosome length.

In addition, there was a significant correlation between both chromosome length and heritability (r = 0.46, p = 0.03), and number of genes per chromosome and heritability (r = 0.61, p = 0.002) in the TS data **(** **Figure 1** **)**. The correlations detected between heritability and chromosome length (r = 0.35, p = 0.11) or between number of genes and heritability (r = 0.38, p = 0.08) for OCD did not reach statistical significance **(** **Figure 2** **)**.

To test individual chromosomes for any significant concentration of heritability beyond that expected by chromosome length, SNP number, or gene number, we calculated the expected proportion of heritability for each chromosome based on the number of SNPs (in our data) as well as the number of genes (from SangerVega) on each chromosome, assuming a polygenic model with a uniform distribution of heritability across the genome. A comparative plot of observed “by chromosome” heritability relative to the expected heritability under the uniform distribution model demonstrated that chromosome 15 harbored a larger proportion of heritability for OCD than expected based on either the number of SNPs or number of genes represented on the chromosome **(Figures S5 and S6)**. When chromosome 15 was removed, a significant correlation between chromosome length and heritability was recovered (r = 0.44, p = 0.05). Greater than expected heritability per chromosome was discovered in the TS data for chromosomes 2, 5, 11, 16 and 20 **(Figures S7and S8)**. In addition, some chromosomes contributed less heritability to OCD than expected due to chromosome length alone. Notably chromosome 6, which houses the HLA locus, did not contribute to overall heritability estimates in OCD or TS.

### Analysis by Minor Allele Frequency (MAF)

**Figure 3:**
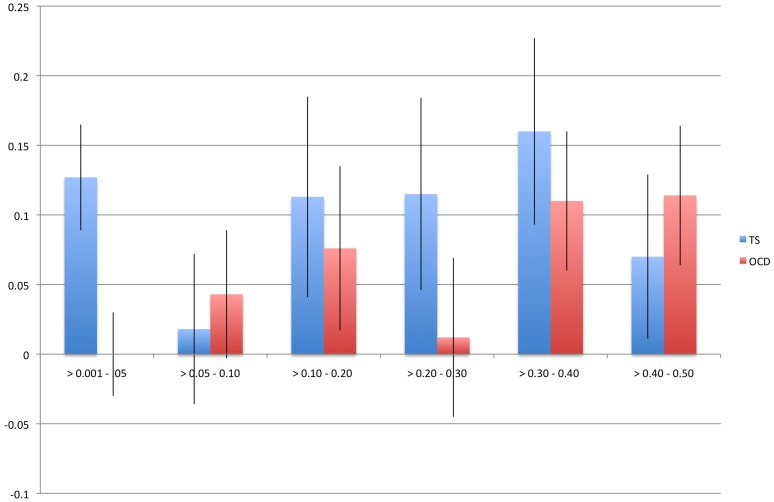
Heritability by minor allele frequency. The x-axis represents all minor allele frequency bins tested while the y-axis represents resultant heritability in a given bin. Blue bars indicate TS and red bars indicate OCD. Error bars are shown.

We identified a significant difference between TS and OCD in the proportion of heritability accounted for by variants with MAF<0.05 **(** **Table 2** **,** **Figure 3** **)**. This result was observed in both the directly genotyped data and imputed data. Using the directly genotyped data, TS SNPs with MAF<0.05 (N = 20,316; 5.3% of all directly genotyped SNPs) represented 21% (0.13, se = 0.04) of the total calculated heritability, while OCD SNPs with MAF<0.05 (N = 19,605; 5.2% of all directly genotyped SNPs) represented 0% (0.000001, se = 0.01) of the total calculated heritability. Similar results were observed using the imputed data, with approximately 30% of the total heritability of TS captured by variants with MAF<0.05 (N = 2,243,744; 30% of all imputed SNPs) and 0% of the total heritability of OCD captured by variants with MAF<0.05 (2,357,568; 30% of all imputed SNPs).

**Table 2:** GWAS and imputed heritability partitioned by minor allele frequency.

Genomic Data Source	MAF	Tourette syndrome			Obsessive-compulsive disorder		
		Number of SNPs (*% of total*)	Heritability (se)	% Heritability	Number of SNPs (*% of total*)	Heritability (se)	% Heritability
GWAS	>0.001–0.05	20,316 (*5.1*)	0.13 (*0.04*)	21%	19,605 (*5.2*)	0.000001 (*0.03*)	0%
	>0.05–0.10	49,445 (*12.5*)	0.02 (*0.05*)	3%	47,976 (*12.8*)	0.04 (*0.05*)	11%
	>0.10–0.20	96,398 (*24.5*)	0.11 (*0.07*)	18%	91,661 (*24.5*)	0.08 (*0.06*)	23%
	>0.20–0.30	81,924 (*20.8*)	0.12 (*0.07*)	20%	77,641 (*20.7*)	0.01 (*0.06*)	3%
	>0.30–0.40	74,393 (*18.9*)	0.16 (*0.07*)	26%	70,193 (*18.7*)	0.11 (*0.05*)	31%
	>0.40–0.50	70,911 (*18.0*)	0.07 (*0.06*)	11%	66,770 (*17.8*)	0.11 (*0.05*)	31%
Imputed	>0.001–0.05	2,243,744 (*28.8*)	0.15 (*0.09*)	31%	2,357,568 (30.0)	0.000001 (*0.06*)	0%
	>0.05–0.50	5,538,943 (*71.2*)	0.34 (*0.10*)	69%	5,492,973 (70.0)	0.32 (*0.12*)	100%

Legend: MAF: minor allele frequency; GWAS: genome-wide association study; se: standard error; SNPs: single nucleotide polymorphisms.

### Analysis by Annotation Classification

In the analysis of directly genotyped data, we found that genic variants accounted for 53% (0.30, se = 0.07; p = 0.008) of the total TS heritability and 40% (h^2^ = 0.15, se = 0.06, p = 0.003) of the total OCD heritability **(Table S7)**. In the analysis of imputed data, parietal lobe eQTLs accounted for 28% (h^2^ = 0.13, se = 0.08; p = 0.03) of the total TS heritability and 29% (h^2^ = 0.09, se = 0.06; p = 0.1) of the total OCD heritability. Cerebellar eQTLs accounted for 35% (h^2^ = 0.11, se = 0.06; p = 0.02) of the total OCD heritability but only 19% (h^2^ = 0.09, se = 0.07; p = 0.1) of the total TS heritability **(** **Table 3** **)**. When the brain eQTLs were further subdivided into parietal “only”, cerebellum “only” and those present in parietal lobe and cerebellum we found that ∼25% of both TS and OCD heritability was accounted for by parietal eQTLs, ∼10% of both TS and OCD heritability was accounted for by eQTLs found in both tissues, and that cerebellar eQTLs again accounted for more heritability (20%) in OCD than in TS (9%) **(Table S8, Figure S9)**. We then tested a final model in which brain eQTLs from cerebellum and parietal tissues were combined into a single “brain-only” partition, and included in the same joint analysis with muscle eQTLs, eQTL found in both brain and muscle, and a non-eQTL partition. In this model, brain eQTLs accounted for 33% (h^2^ = 0.16, se = 0.10, p = 0.06) of the total TS heritability and 59% (h^2^ = 0.19, se = 0.08, p = 0.009) of the total heritability for OCD. Skeletal muscle eQTLs accounted for 25% (h^2^ = 0.12; se = 0.10; p = 0.1) of the total TS heritability and 25% (h^2^ = 0.08; se = 0.09; p = 0.2) of the total heritability for OCD. The overlapping set of eQTLs identified in both muscle and brain accounted for 8% heritability in TS (h^2^ = 0.04; se = 0.08; p = 0.3) and 0% (h^2^ = 0.0000001; se = 0.06; p = 0.5) of total OCD heritability. Finally, the remaining non-eQTL portion of SNPs accounted for only 34% (h^2^ = 0.16; se = 0.16; p = 0.2) of TS heritability and 16% (h^2^ = 0.05; se = 0.08; p = 0.3) of OCD heritability **(Table S9, Figure S10)**.

**Table 3:** Heritability of Tourette syndrome and obsessive-compulsive disorder partitioned by SNPs annotated as expression quantitative trait loci in parietal cortex and cerebellum.

Brain tissue	Partition	Number of SNPs	Proportion of total SNPs	Tourette syndrome		Obsessive-compulsive disorder	
				Heritability	Proportion of total heritability estimate	Heritability	Proportion of total heritability estimate
				(se)		(se)	
Parietal Lobe	eQTL	383,052	5%	0.13	28%	0.09	29%
				(0.07)		(0.06)	
	Non-eQTL	7,261,032	95%	0.33	72%	0.22	71%
				(0.11)		(0.09)	
Cerebellum	eQTL	459,415	6%	0.09	19%	0.11	35%
				(0.07)		(0.06)	
	Non-eQTL	7,044,239	94%	0.38	81%	0.2	65%
				(0.12)		(0.09)	

Legend: se: standard error; SNPs: single nucleotide polymorphisms; eQTL: Expression Quantitative Trait Locus.

### Age of Onset (OCD only)

It has been observed that early-onset OCD is more heritable (h^2^ = 45–65%) than adult-onset OCD (h^2^ = 27–47%) [16], [46]. To test this hypothesis in our data, the OCD sample was divided by age of diagnosis into early-onset (<16 years), yielding 732 case samples with early-onset OCD, and 267 case samples with adult-onset OCD. The heritability for early-onset OCD was 0.43 (se = 0.10) and for adult-onset was 0.26 (se = 0.24)**(** **Table 1** **)**.

## Discussion

GCTA has now been applied to a number of complex traits, including TS and OCD **(Table S10)**. Results from all of these analyses show that common interrogated variants account for a significant proportion of heritability estimated from twin and family studies [4]–[8], . Depending on the phenotype and original literature estimates, the proportion of heritability explained by common variation varies across different disorders from essentially all estimated heritability, as observed in autism, multiple sclerosis and von Willebrand's factor, to roughly half of the estimated heritability, as observed in height, schizophrenia, and type 1 diabetes. This study represents the first effort to use genome-wide genotype data to determine the heritability of two related neuropsychiatric disorders, OCD and TS. The narrow-sense heritability of each disorder (h^2^_GCTA_ = 0.58 for TS and 0.37 for OCD) correspond well with previously reported heritability estimates from family and twin studies [17], [19], [20], [21], [22], [23]–[25], [26], [27], [28], [29], [49] suggesting that there is little, if any, heritability “missing” (i.e., unassayed). While previous TS and OCD GWAS have been underpowered to identify individual susceptibility variants with modest effect sizes, based on these results, future GWAS in much larger samples should identify a large number of true TS and OCD disease variants.

The difference between the heritability estimates calculated from imputed and directly genotyped data was not significant. However, the imputed heritability estimates were slightly but consistently lower compared to the estimates generated from the directly genotyped data. While we employed strict r^2^ thresholds, the dosage format of imputed data prevented it from being subjected to the same strict Hardy-Weinberg thresholds as the directly genotyped data. Therefore this small decrease in measured heritability may reflect additional noise in the imputed data contributed by lower quality SNPs. Alternatively, the decrease may reflect the possibility that even with very stringent QC some minor residual technical artifacts may have remained in the directly genotyped data. Perhaps most interesting though, is the observation that the imputed data did not show a significant *increase* in heritability, even with a substantial increase in the number of interrogated variants, suggesting that the directly genotyped data alone sufficiently captured the narrow-sense heritability present in SNP level data.

We identified a significant genetic correlation between TS and OCD of 0.41 (se = 0.15). This estimate of genetic overlap is smaller than that observed for schizophrenia/bipolar disorder (0.68±0.04), but similar to that of bipolar disorder/major depressive disorder (0.47±0.06) and schizophrenia/major depressive disorder (0.43±0.06) [50]. While this result suggests there is some degree of shared heritability between the two disorders, the standard error of the genetic correlation was large. In addition, the presence of overlapping co-morbidity between TS and OCD in both samples (13% co-morbid TS or tics in the OCD sample, 43% co-morbid OCD in the TS sample) may have inflated the correlation further. After removing all TS and OCD cases with documented co-morbid OCD or TS, respectively, the subsequent cleaner, but underpowered analysis yielded a genetic correlation of 0.50 (se = 0.29) which is very similar to the initial correlation of r = 0.41. It is important to note, however, that some cases with missing co-morbidity data may have contributed residual co-morbidity to this sensitivity analysis. Therefore, the bivariate genetic correlation may still be an overestimate, and should be interpreted with caution.

We went on to examine the genomic distribution of liability by partitioning the heritability by chromosome. We found that the additive heritability estimated by chromosome for either OCD or TS was not significantly different from the cumulative univariate heritability calculated by using all data together. This served as an additional quality control check and confirmed the absence of residual LD between chromosomes, which can arise in a sample with cryptic relatedness or population substructure [51]. We examined the relationship between chromosome length and proportion of heritability detected, which also provides insight into the distribution of risk alleles throughout the genome and helps to characterize the polygenic contribution to risk. We found evidence, in both TS and OCD, of a highly polygenic architecture, as demonstrated by the significant correlation between chromosomal length and heritability. In addition, the observation that individual chromosomes in both phenotypes contributed to heritability disproportionately suggest these chromosomes may harbor loci with larger effect sizes on a polygenic background of small effect susceptibility variants distributed equally throughout the genome.

The initial correlation between OCD heritability and chromosome length increased substantially after removal of chromosome 15 **(Tables S5 and S6)**. This increase in correlation is quite similar to the increase in the correlation between chromosome length and heritability reported for multiple sclerosis (MS) [48] upon removal of chromosome 6 (r = 0.45), suggesting that chromosome 15 may contribute to the heritability of OCD much to the same degree that chromosome 6 contributes to the heritability of MS [48]. Regions of chromosome 15 have been identified as linkage signals for OCD across multiple populations [52], [53]. Additionally, genes within the imprinted genomic region chr15q11-13 have been reproducibly associated with repetitive behaviors, obsessive compulsive behaviors, and autism [54], [55], [56], [57]. Together these findings continue to implicate chromosome 15 in the development of OCD.

Of note, essentially no heritability for either OCD or TS was observed on chromosome 6, which encodes both the HLA and histone gene clusters. This absence of heritability within the MHC region is relevant to these two phenotypes, since an autoimmune etiology for both OCD and TS has been proposed, based on similarities between these two disorders and the acute neuropsychiatric presentation of patients with Sydenham chorea in the setting of acute rheumatic fever and triggered by Group A streptococcal infection [58]. While an immune-mediated mechanism could still arise from genetic loci outside of the HLA locus, our result is in stark contrast to schizophrenia, where the strongest GWAS signal is observed in HLA, suggesting this disorder has an immune-mediated component [59].

In an effort to further understand the genomic architecture of OCD and TS, we performed exploratory analyses of heritability across the MAF spectrum. By running all MAF bins together in a single REML analysis, we partitioned the effects of LD across each bin, as Lee et al (2012) previously demonstrated through simulation that this approach restricts the effects of LD between bins and reflects expected heritability per bin based on simulated risk allele distributions. For OCD, *no* heritability was captured by SNPs with MAF<5%, while the majority of the heritability detected was due to those SNPs with MAF>30%. In contrast, for TS, 21% of the total heritability was captured by SNPs with MAF less than 5% with the remaining bulk of the heritability shared approximately equally among alleles with MAF between 0.10–0.50. Analysis of imputed data confirmed these findings and showed that SNPs with MAF<0.05 accounted for 30% of the total TS heritability and 0% of the total OCD heritability. To ensure that the difference between TS and OCD rare SNP heritability estimates were not due to subtle population substructure in the TS sample, we conducted an additional analysis which further partitioned the MAF<5% bin by chromosome. We then compared the estimate of heritability calculated by summing each chromosome (h^2^ = 12.3, se = 0.08) to the estimate of heritability based on all MAF<5% SNPs in a single analysis (h^2^ = 12.7, se = 0.04) and found no significant difference. If population substructure was present in the TS sample and was a source of bias contributing to the increased heritability identified in the rare bin, we would have expected to see inflation of the heritability estimate due to LD between chromosomes when partitioned by chromosome and then summed [51]. We can therefore reject the hypothesis that the rare variant heritability in TS is due to population substructure.

The observation that TS and OCD have such different patterns for heritability estimated across the MAF spectrum points to the value that such analyses may provide for illuminating genetic architecture. There is clearly support for analysis of rare variants and follow-up sequencing in TS given the contribution to heritability observed for SNPs with MAF<0.05. The observations in OCD are also intriguing with respect to questions on the set of genetic models that would be consistent with heritability being concentrated among variants with high MAF. Are such patterns consistent with particular models for the age of a disorder, or perhaps with aspects of the evolutionary history of contributing risk alleles? It will also be important to investigate whether such analyses applied to other disorders will reveal a full continuum with respect to the proportion of phenotypic variance attributable to variants across the MAF spectrum or something more discrete with overall patterns more similar to OCD at one end and TS at the other. Replication analysis with larger samples and additional phenotypes will undoubtedly shed more light on the analysis presented here.

We partitioned SNPs annotated as brain (parietal and cerebellum) and muscle eQTLs in an effort to concentrate heritability within smaller putatively functional classes of testable variants [60]. Taken together, these results suggest a substantial contribution to overall heritability by SNPs annotated as brain eQTLs for both TS and OCD. However, it is important to note that several limitations of experimental power, including power to detect eQTLs across tissues, and power to estimate heritability within our samples, resulted in large standard errors. Cautious interpretation of these exploratory analyses finds that the “brain-only” eQTL partition in OCD provides the only statistically significant estimate of heritability (h^2^ = 0.19, se = 0.08, p = 0.009) in a joint analysis with an additional non-brain tissue (muscle), although the TS “brain-only” partition approaches significance (h^2^ = 0.16, se = 0.10, p = 0.06) (Table S9; Figure S8). The result is intriguing especially considering that the non-eQTL partition contained over 6.5 million SNPs, approximately twelve times the number of SNPs contained in the brain-only eQTL partition. These findings are preliminary and will require replication. Nevertheless, when interpreted in the context of additional recent studies showing specific enrichment of brain eQTLs in top GWAS signals from neuropsychiatric phenotypes, our results suggest that further study of the role of brain eQTLs in TS and OCD is warranted [61], [38].

Our results examining the heritability of childhood-onset OCD are in line with previous studies that suggest a higher heritability for childhood-onset OCD than for adult onset OCD. However, because of the smaller sample sizes due to splitting the OCD sample into two groups based on age of onset, the 95% confidence intervals for childhood-onset and adult-onset OCD overlap, and are not significantly different from each other. With increased sample sizes it may be possible to confirm these observed heritability differences and to obtain more precise estimates of the relative heritability of child and adult onset OCD.

Our results explain essentially all of the heritability of TS and a majority of the heritability of OCD established by twin and family studies. One factor that may have contributed to the significant proportion of heritability explained by our results is the ascertainment strategy employed to collect the samples. As Klei and colleagues (2012) elegantly demonstrated, heritability estimated from samples belonging to multiplex families can be greater than those generated by samples belonging to simplex families. This phenomenon is most likely a matter of increased polygenic load reflected in the multiplex samples, as opposed to differing allelic architectures [6]. Approximately 30% of the TS cases used in this analysis came from families with more than one affected individual. Replication of these results in other samples and populations will be needed to further confirm the heritability estimates and partition estimates presented here.

In conclusion, this study provides substantial evidence that both TS and OCD are highly heritable, polygenic, and that a significant majority of the heritability of both disorders is captured by GWAS SNP variants. Using both directly genotyped and imputed data, we also provide evidence of allelic architecture differences between TS and OCD. Specifically, we identified a significant contribution from rare variants in the genomic architecture of TS that appears to be absent from the architecture of OCD. Our results also provide additional evidence of a prominent role for chromosome 15 in OCD liability and possible concentration of TS liability on chromosomes 2, 5, 11, 12, 16 and 20. We also find that brain eQTLs concentrate a significant proportion of the heritability present in TS and OCD. It is unlikely that the differences in genetic architecture between TS and OCD are due to incomplete matching during QC or other, unknown, technical biases, as all cases were genotyped with identical technology, shared the same control set, and were imputed together. Taken together, these results advance our understanding of the overlapping and non-overlapping genomic architectures of TS and OCD and suggest that non-overlapping elements of the architecture of each phenotype may be a limiting factor in the genetic relationship between them. Moreover, these results may be used to inform priorities for future studies of both disorders. For example, given the apparent contribution of rare variants to the heritability of TS, DNA sequencing may be a particularly informative analysis, whereas larger sample sizes and additional GWAS is likely to identify the majority of susceptibility variants for both disorders. Future studies aimed at understanding the genetic control of shared neurocircuitry in TS and OCD may be most well powered by testing the association of shared genetic risk (i.e., common polygenic brain eQTLs) with a well-defined quantitative neurobiological endophenotype. Studies such as the one presented here continue to highlight the value of “big picture” analyses, which provide insight into the genetic landscape of a phenotype, as a necessary and intelligent complement to the mapping of specific risk variants.

## Supporting Information

Figure S1 Q-Q plot of the distribution of p-values for all directly genotyped SNPs in the “control-control” logistic regression analysis in which platform was substituted for phenotype. The top 5 principal components were used as covariates in the analysis. We observed no deviation from the expected distribution under the null hypothesis of no association.
(JPG)

Figure S2 The distribution of pi-hat (empirical estimates of relatedness) among TS cases. A pi-hat threshold of 0.05 was implemented for all analyses.
(JPG)

Figure S3 The distribution of pi-hat (empirical estimates of relatedness) among OCD cases. A pi-hat threshold of 0.05 was implemented for all analyses.
(JPG)

Figure S4 The distribution of pi-hat (empirical estimates of relatedness) among controls. A pi-hat threshold of 0.05 was implemented for all analyses.
(JPG)

Figure S5 The x-axis of Figure 5 shows the difference between the actual OCD heritability calculated per chromosome and the expected heritability calculated per chromosome based on the proportion of genes represented by the given chromosome. Each grey bar represents a chromosome and the error bars shown represent the error in the actual heritability estimate. The only chromosome showing significant deviation from expectation is chromosome 15.
(PDF)

Figure S6 The x-axis of Figure 6 shows the difference between the actual OCD heritability calculated per chromosome and the expected heritability calculated per chromosome based on the proportion of SNPs represented by the given chromosome. Each grey bar represents a chromosome and the error bars shown represent the error in the actual heritability estimate. The only chromosome showing significant deviation from expectation is chromosome 15.
(PDF)

Figure S7 The x-axis of Figure 7 shows the difference between the actual TS heritability calculated per chromosome and the expected heritability calculated per chromosome based on the proportion of genes represented by the given chromosome.
(PDF)

Figure S8 The x-axis of Figure 8 shows the difference between the actual TS heritability calculated per chromosome and the expected heritability calculated per chromosome based on the proportion of SNPs represented by the given chromosome. Each grey bar represents a chromosome and the error bars shown represent the error in the actual heritability estimate. Chromosomes 2, 5, 16 and 20 show increased heritability compared to expectation based on both proportion of genes and proportion of SNPs.
(PDF)

Figure S9 Figure displays the eQTL annotation based bins including 1) a “parietal-only” bin consisting of eQTLs identified in parietal cortex and not in cerebellum, 2) a “cerebellum-only” bin consisting of eQTLs identified in cerebellum and not in parietal cortex, and a “parietal and cerebellum” bin consisting of eQTLs identified in both cerebellum and parietal cortex. Finally, a non-eQTL partition was included.
(PDF)

Figure S10 Figure displays the eQTL annotation-based bins including 1) a “brain-only” bin consisting of eQTLs identified in parietal cortex or cerebellum and not in muscle, 2) a “muscle-only” bin consisting of eQTLs identified in muscle and not in parietal cortex or cerebellum, and a “brain and muscle” bin consisting of eQTLs identified in muscle and either cerebellum, parietal cortex, or both. Finally, a non-eQTL partition was included. The asterisk represents a significant p-value of p = 0.009.
(PDF)

Methods S1 Description of additional methods used in the quality control of samples and SNPs for GCTA analysis. Additionally, a brief description of the identification of eQTL. Finally we provide analytic details of the calculation of heritability on the sibling recurrence risk scale.
(DOC)

Table S1 Effects of differing pi-hat thresholds on Tourette Syndrome and OCD heritability estimates. Pi-hat refers to the proportion of alleles shared IBD and thus represents a relatedness threshold required for each analysis.
(DOC)

Table S2 Control-control analysis with differing QC thresholds. Table showing changes to the control-control heritability estimate based on differing filtering approaches to the data. Numbers in each cell represent the number of SNPs filtered based on each threshold. MAF = minor allele frequency. Diff SNP Missing = genotypic differential missingness rate. HWD = SNPs with significant deviation (p<0.05) from Hardy Weinberg Equilibrium. SNP Call Rate = Genotyping call rate per sample. Platform Effect SNP = SNPs with significant platform effects. Total # SNPs = Total number of SNPs surviving QC and used in heritability analysis. Total # Sample = Total number of subjects surviving QC and used in heritability analysis. Heritability (se) = Heritability point estimate and standard error of the estimate. P-value = likelihood ration test generated p-value for significance of heritability estimate.
(DOC)

Table S3 Heritability for Tourette syndrome, obsessive-compulsive disorder, and early onset obsessive-compulsive disorder at a range of reported population prevalence rates.
(DOC)

Table S4 Heritability in terms of sibling recurrence risk (λ) for Tourette syndrome, obsessive-compulsive disorder, and early onset obsessive-compulsive disorder at a range of population prevalences. The title λ_1st-GCTA_ refers to the risk to first degree relatives calculated from the given population prevalence and GCTA based heritability estimate. The title λ_1st-lit_ refers to the risk to first degree relatives calculated from the given population prevalence and the heritability estimates from the literature cited in the main text of the paper.
(DOC)

Table S5 Tourette Syndrome heritability partitioned by chromosome. Heritability estimates given for each chromosome for both directly genotyped and imputed data. P-values calculated with a likelihood ratio test are also included; * indicates p-values significant after Bonferroni correction.
(DOC)

Table S6 Obsessive-compulsive disorder heritability partitioned by chromosome. Heritability estimates given for each chromosome for both directly genotyped and imputed data. P-values calculated with a likelihood ratio test are also included; * indicates p-values significant after Bonferroni correction.
(DOC)

Table S7 GWAS estimated heritability partitioned by genic regions. Heritability estimates for TS and OCD partitioned based on genic annotation. “Genic” includes all coding, intronic, 3′UTR and 5′UTR SNPs. Intergenic is defined as not otherwise genic. The number of SNPs (proportion of total SNPs), heritability, and proportion of total heritability is given for TS and OCD.
(DOC)

Table S8 Partitioning analysis of heritability based on brain eQTL annotations. Partitions include eQTLs identified in cerebellum only, in parietal cortex only, in both parietal cortex and cerebellum, and non-eQTL SNPs.
(DOC)

Table S9 Partitioning analysis of heritability based on brain and skeletal muscle eQTL annotations. Partitions include eQTLs identified in brain only, in muscle only, in both brain and muscle, and non-eQTL SNPs.
(DOC)

Table S10 Proportion heritability and correlation with chromosome length for all phenotypes analyzed with GCTA. Table includes data from representative account of GCTA publications with respective reference, phenotype studied, proportion of total twin/family study heritability estimated by GCTA analysis, correlations reported for heritability by chromosome and chromosome length, adjusted correlation reported for heritability by chromosome and chromosome length (upon removal of outliers).
(DOC)
