# Supplementary figures and images for: Partitioning the Heritability of Tourette Syndrome and Obsessive Compulsive Disorder Reveals Differences in Genetic Architecture
Source: PLoS Genet. 2013 Oct 24;9(10):e1003864. doi: 10.1371/journal.pgen.1003864 (PMC3812053; doi:10.1371/journal.pgen.1003864)

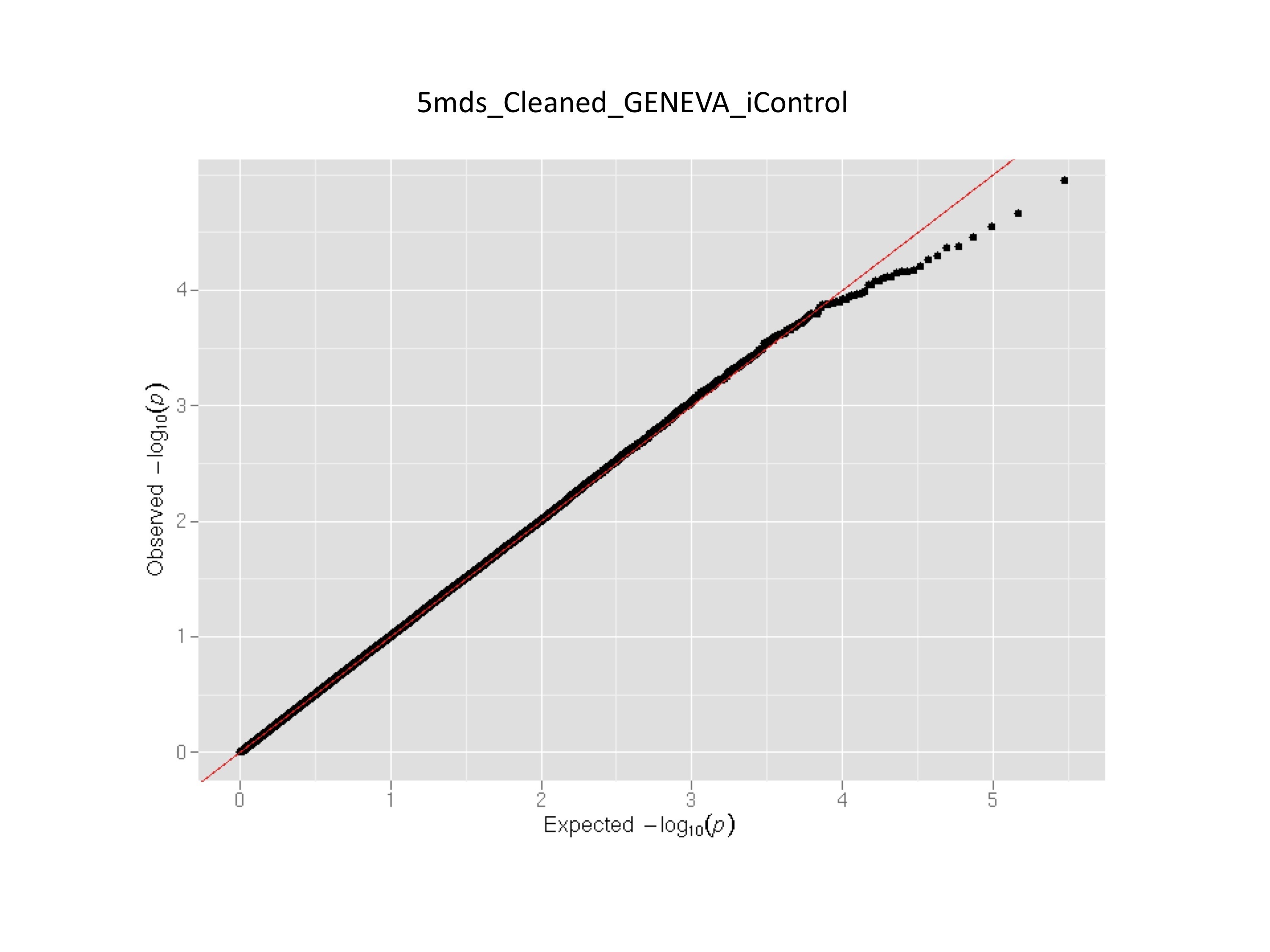

Supplement: Figure S1 — Q-Q plot of the distribution of p-values for all directly genotyped SNPs in the “control-control” logistic regression analysis in which platform was substituted for phenotype. The top 5 principal components were used as covariates in the analysis. We observed no deviation from the expected distribution under the null hypothesis of no association. (JPG) [file pgen.1003864.s001.jpg]

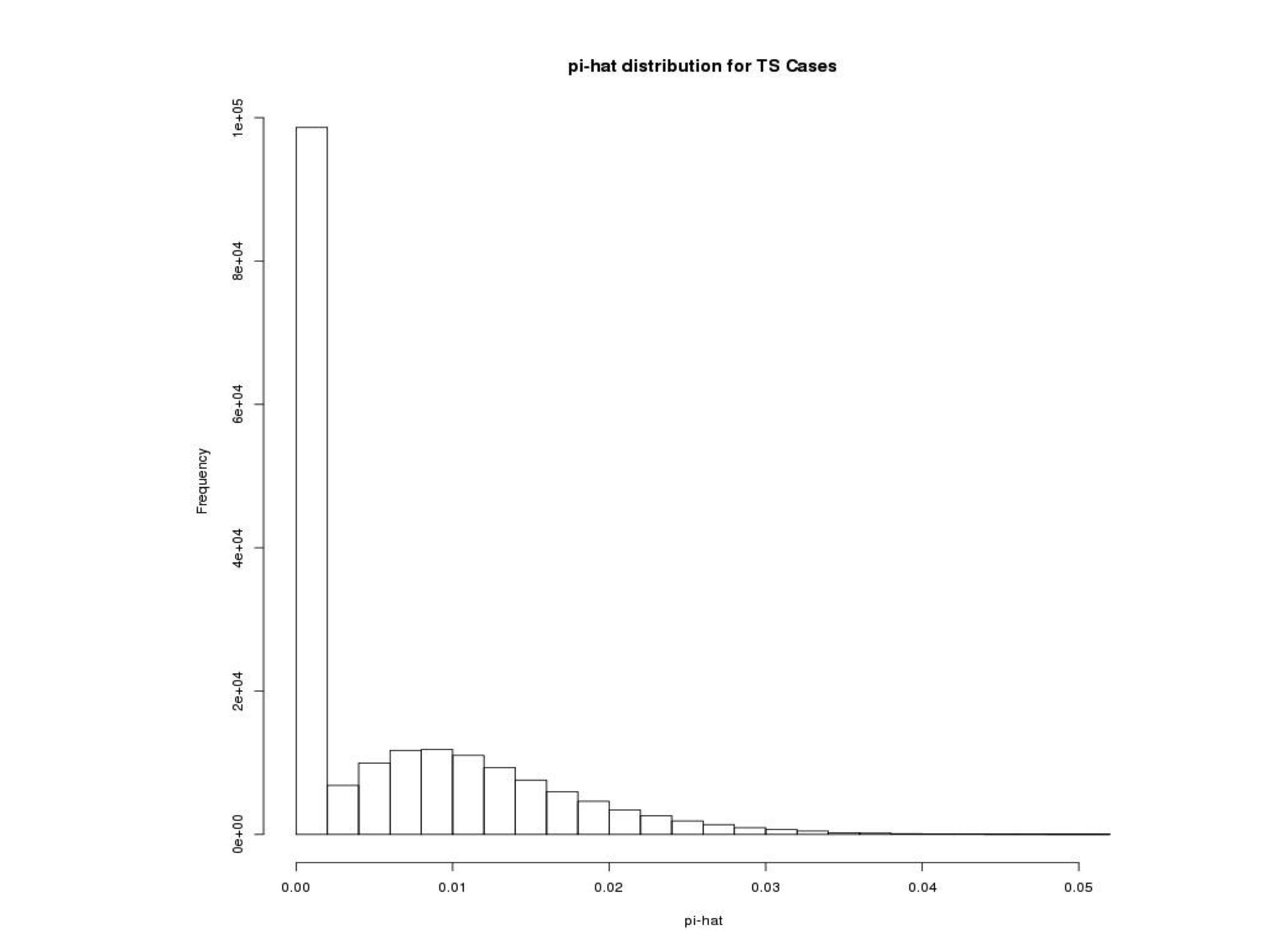

Supplement: Figure S2 — The distribution of pi-hat (empirical estimates of relatedness) among TS cases. A pi-hat threshold of 0.05 was implemented for all analyses. (JPG) [file pgen.1003864.s002.jpg]

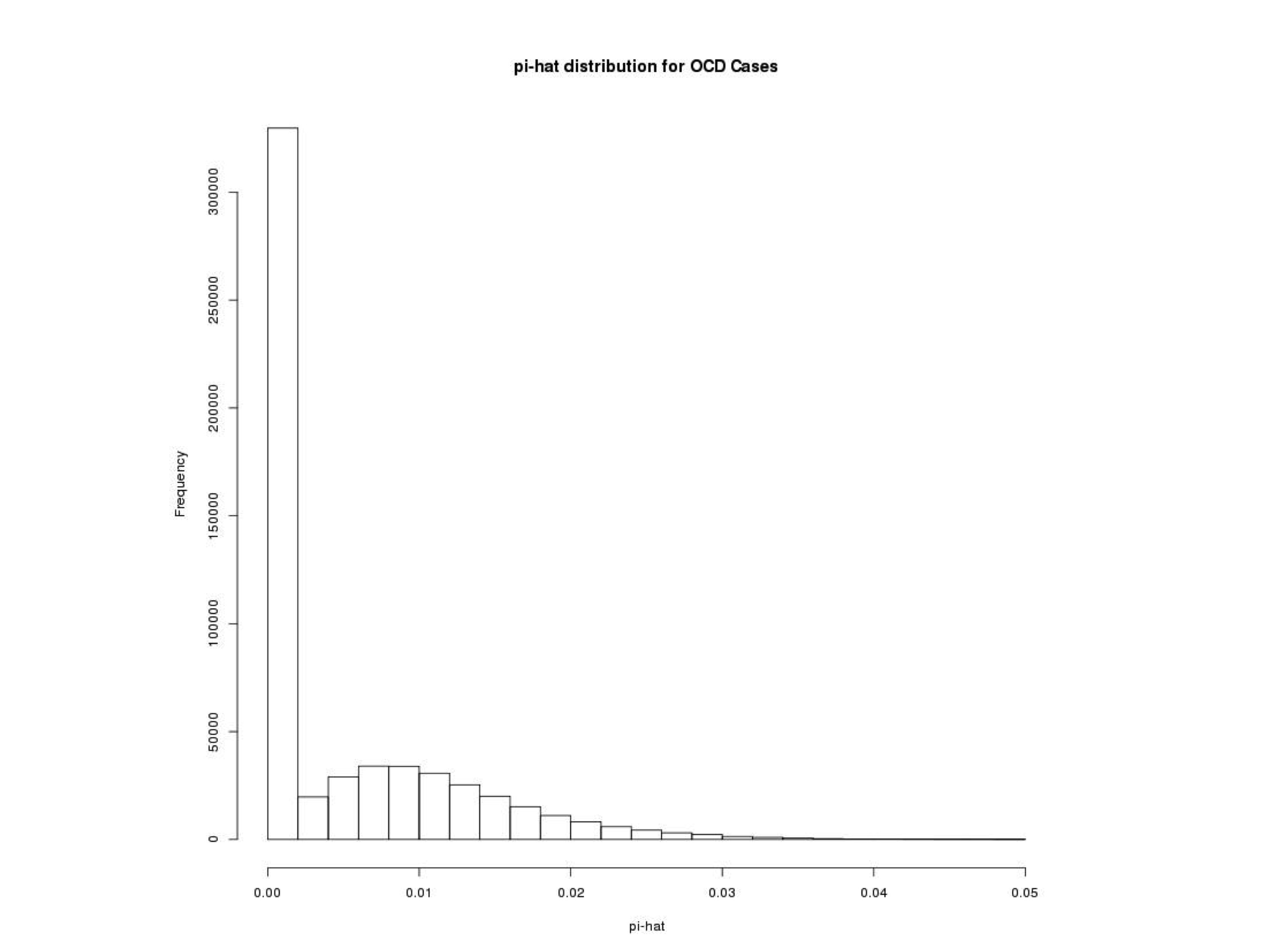

Supplement: Figure S3 — The distribution of pi-hat (empirical estimates of relatedness) among OCD cases. A pi-hat threshold of 0.05 was implemented for all analyses. (JPG) [file pgen.1003864.s003.jpg]

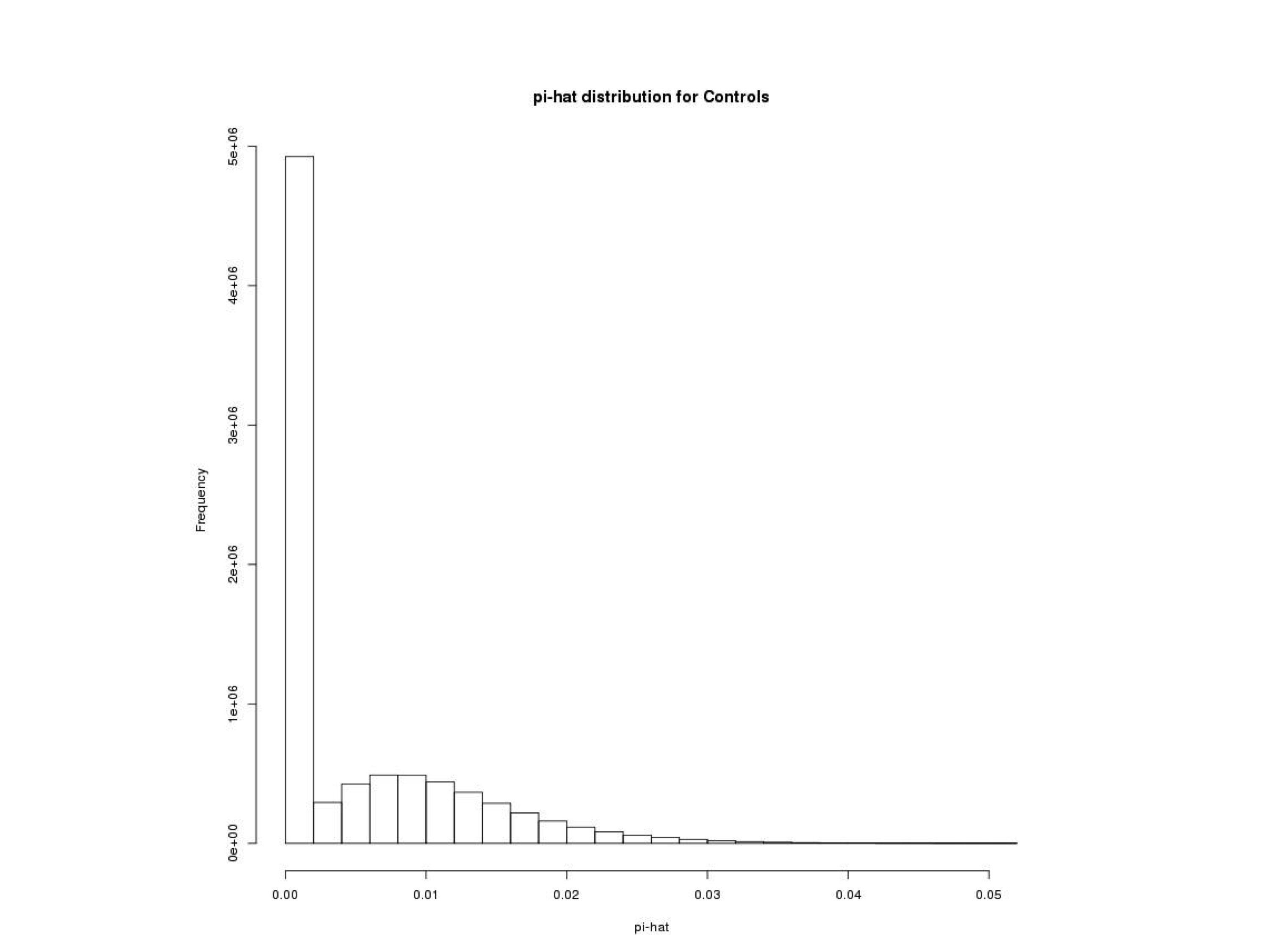

Supplement: Figure S4 — The distribution of pi-hat (empirical estimates of relatedness) among controls. A pi-hat threshold of 0.05 was implemented for all analyses. (JPG) [file pgen.1003864.s004.jpg]

# Difference From Expected Heritability By Chromosome

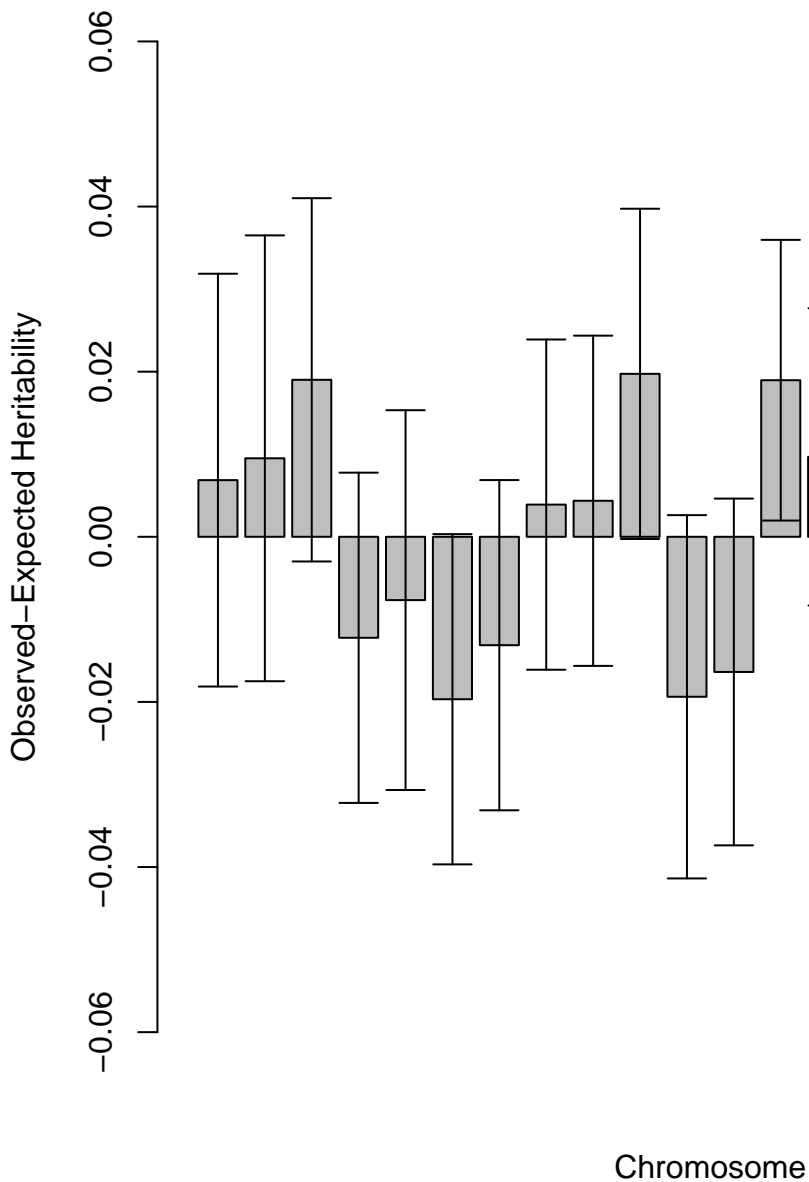

Supplement: Figure S5 — The x-axis of Figure 5 shows the difference between the actual OCD heritability calculated per chromosome and the expected heritability calculated per chromosome based on the proportion of genes represented by the given chromosome. Each grey bar represents a chromosome and the error bars shown represent the error in the actual heritability estimate. The only chromosome showing significant deviation from expectation is chromosome 15. (PDF) [file pgen.1003864.s005.pdf]

# Difference From Expected Heritability By Chromosome

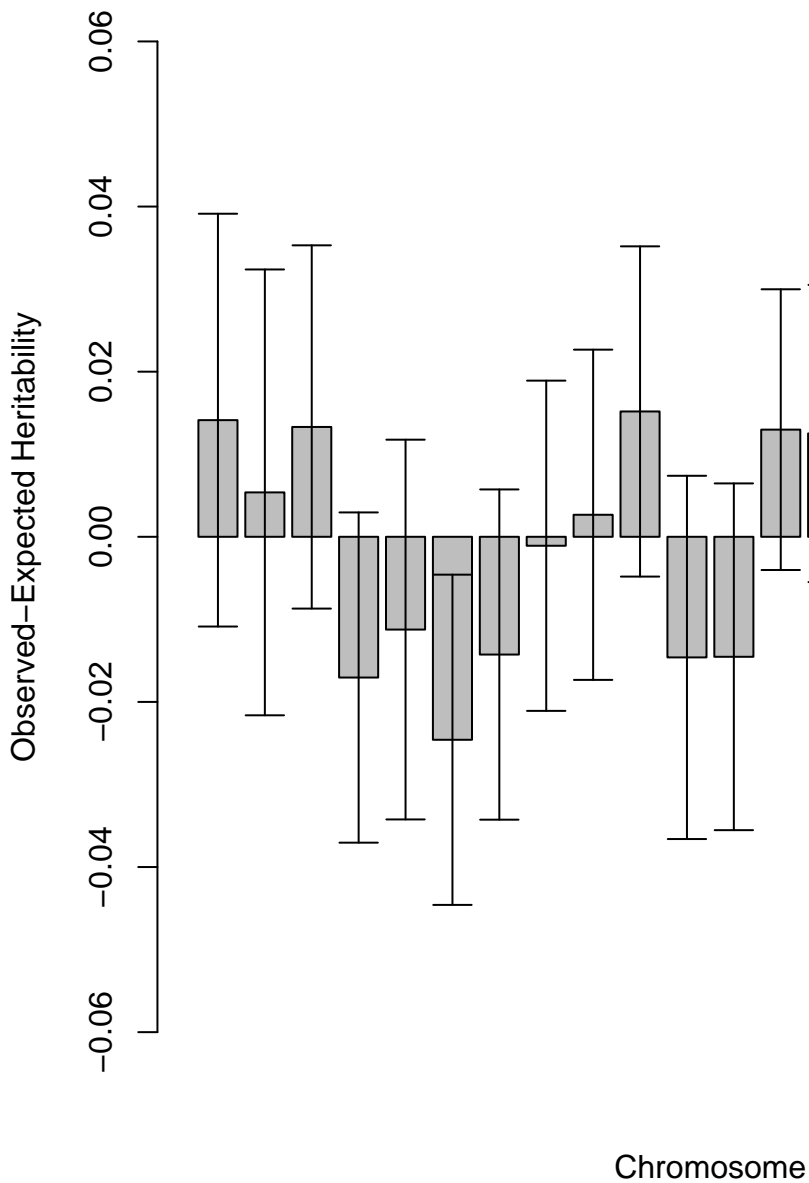

Supplement: Figure S6 — The x-axis of Figure 6 shows the difference between the actual OCD heritability calculated per chromosome and the expected heritability calculated per chromosome based on the proportion of SNPs represented by the given chromosome. Each grey bar represents a chromosome and the error bars shown represent the error in the actual heritability estimate. The only chromosome showing significant deviation from expectation is chromosome 15. (PDF) [file pgen.1003864.s006.pdf]

# Difference From Expected Heritability By Chromosome

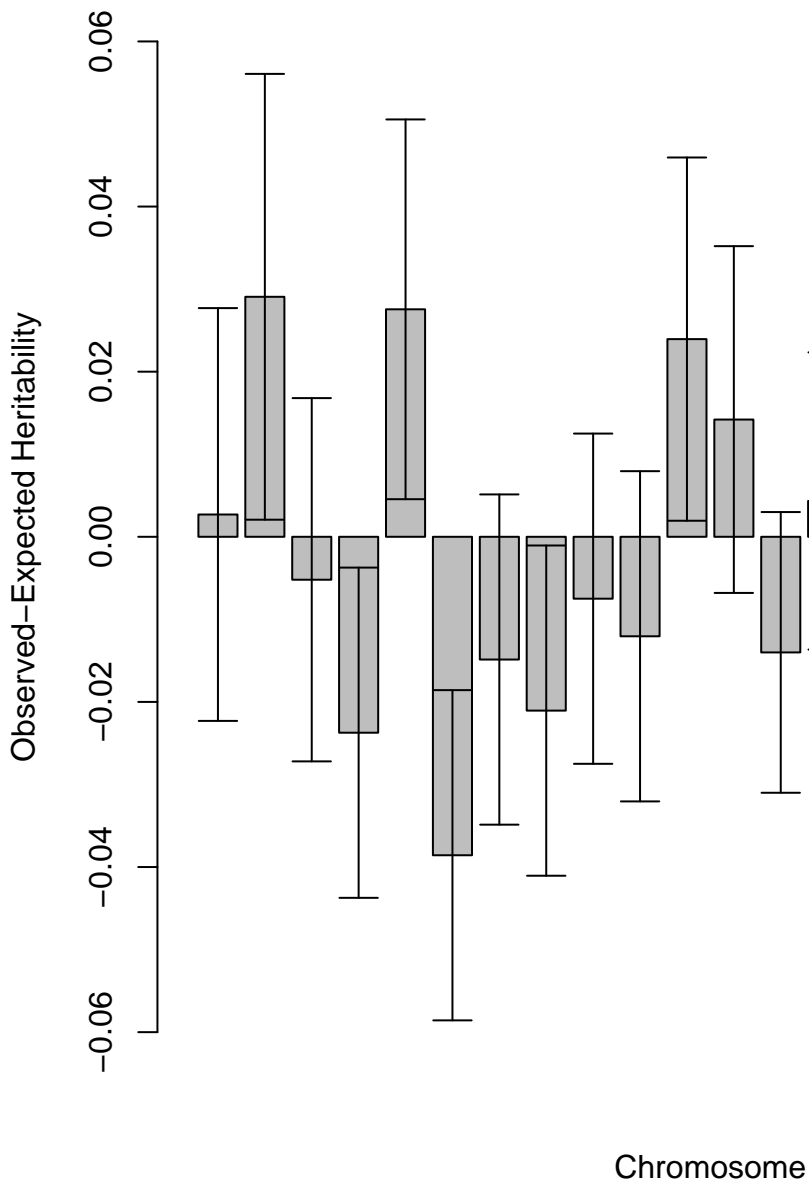

Supplement: Figure S8 — The x-axis of Figure 8 shows the difference between the actual TS heritability calculated per chromosome and the expected heritability calculated per chromosome based on the proportion of SNPs represented by the given chromosome. Each grey bar represents a chromosome and the error bars shown represent the error in the actual heritability estimate. Chromosomes 2, 5, 16 and 20 show increased heritability compared to expectation based on both proportion of genes and proportion of SNPs. (PDF) [file pgen.1003864.s008.pdf]

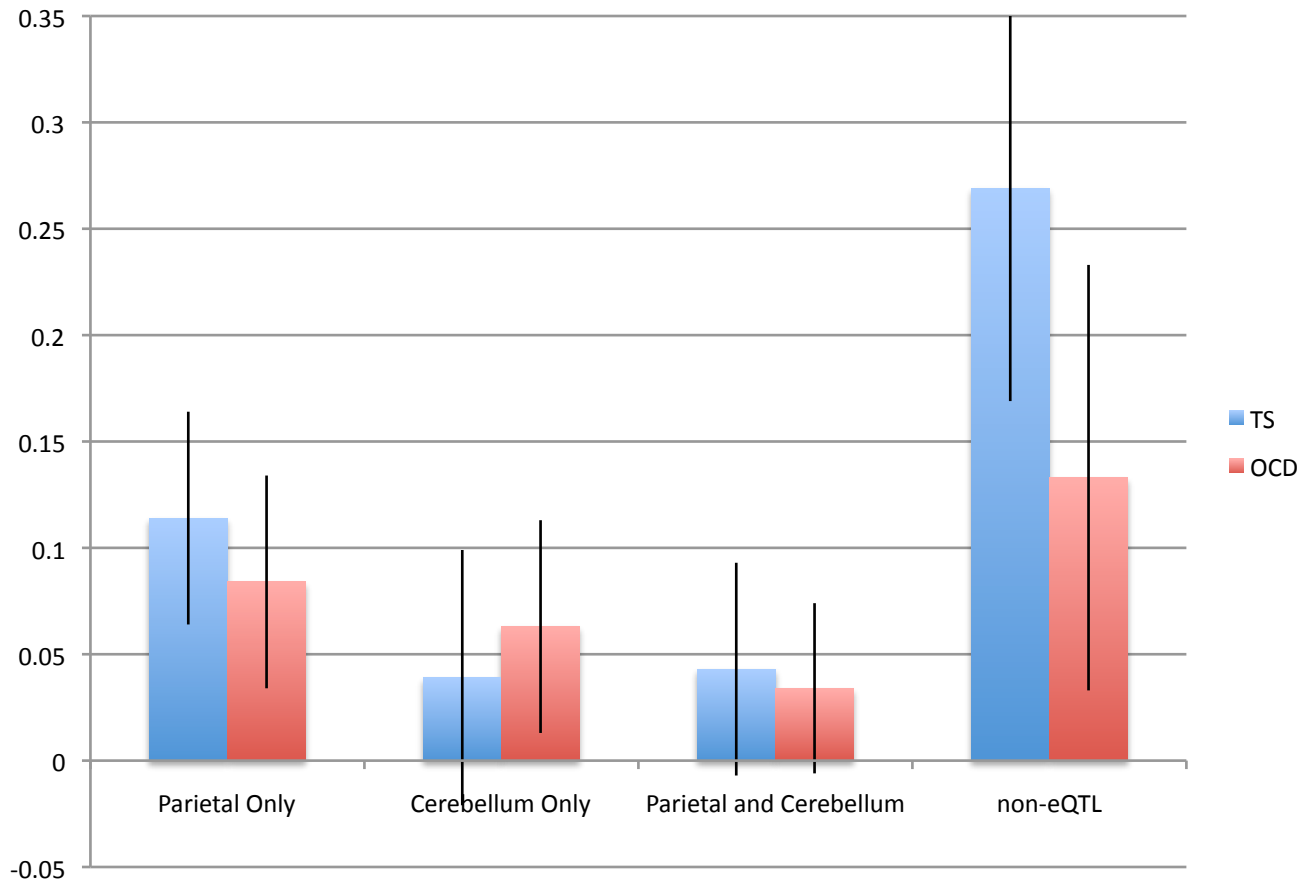

Supplement: Figure S9 — Figure displays the eQTL annotation based bins including 1) a “parietal-only” bin consisting of eQTLs identified in parietal cortex and not in cerebellum, 2) a “cerebellum-only” bin consisting of eQTLs identified in cerebellum and not in parietal cortex, and a “parietal and cerebellum” bin consisting of eQTLs identified in both cerebellum and parietal cortex. Finally, a non-eQTL partition was included. (PDF) [file pgen.1003864.s009.pdf]

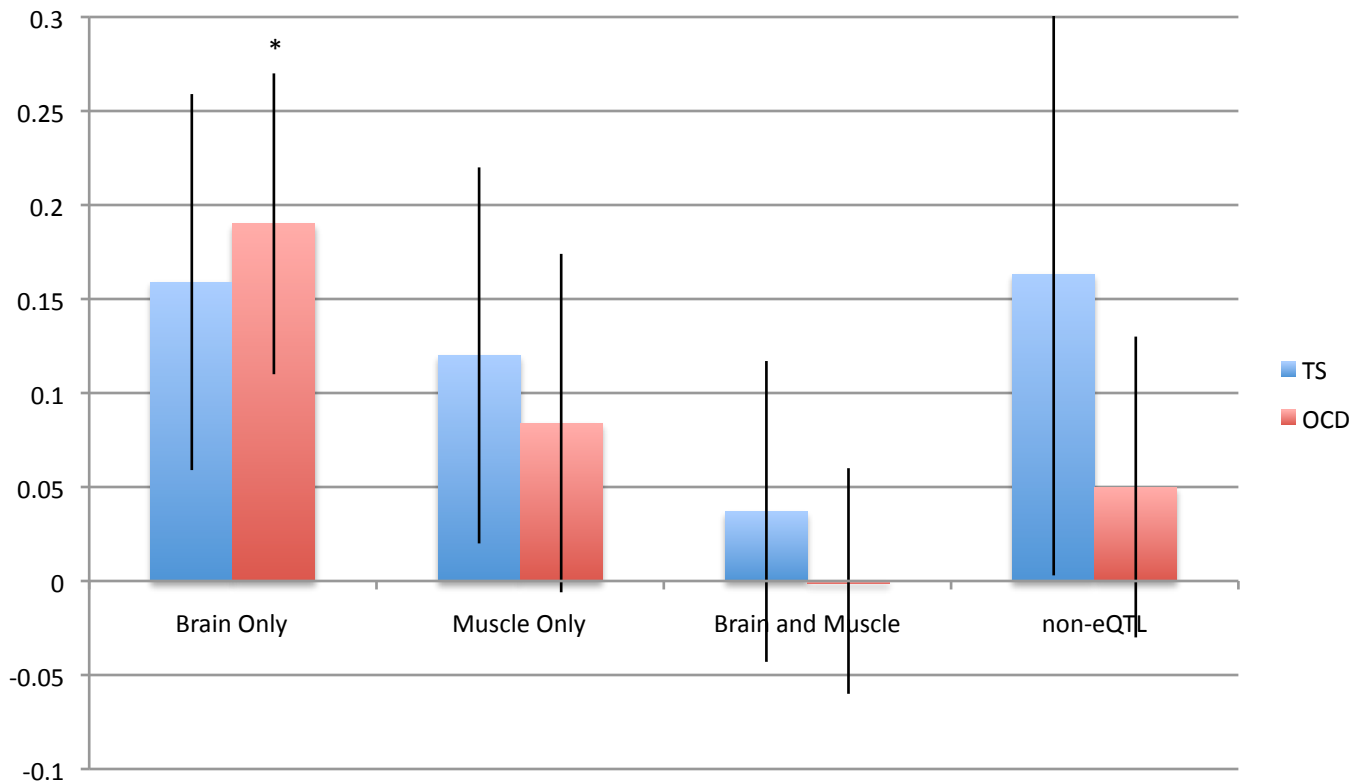

Supplement: Figure S10 — Figure displays the eQTL annotation-based bins including 1) a “brain-only” bin consisting of eQTLs identified in parietal cortex or cerebellum and not in muscle, 2) a “muscle-only” bin consisting of eQTLs identified in muscle and not in parietal cortex or cerebellum, and a “brain and muscle” bin consisting of eQTLs identified in muscle and either cerebellum, parietal cortex, or both. Finally, a non-eQTL partition was included. The asterisk represents a significant p-value of p = 0.009. (PDF) [file pgen.1003864.s010.pdf]
