# Supplementary material for: Partitioning the Heritability of Tourette Syndrome and Obsessive Compulsive Disorder Reveals Differences in Genetic Architecture
Source: PLoS Genet. 2013 Oct 24;9(10):e1003864. doi: 10.1371/journal.pgen.1003864 (PMC3812053; doi:10.1371/journal.pgen.1003864)
Supplement: Figure S7 — The x-axis of Figure 7 shows the difference between the actual TS heritability calculated per chromosome and the expected heritability calculated per chromosome based on the proportion of genes represented by the given chromosome. (PDF) [file pgen.1003864.s007.pdf]

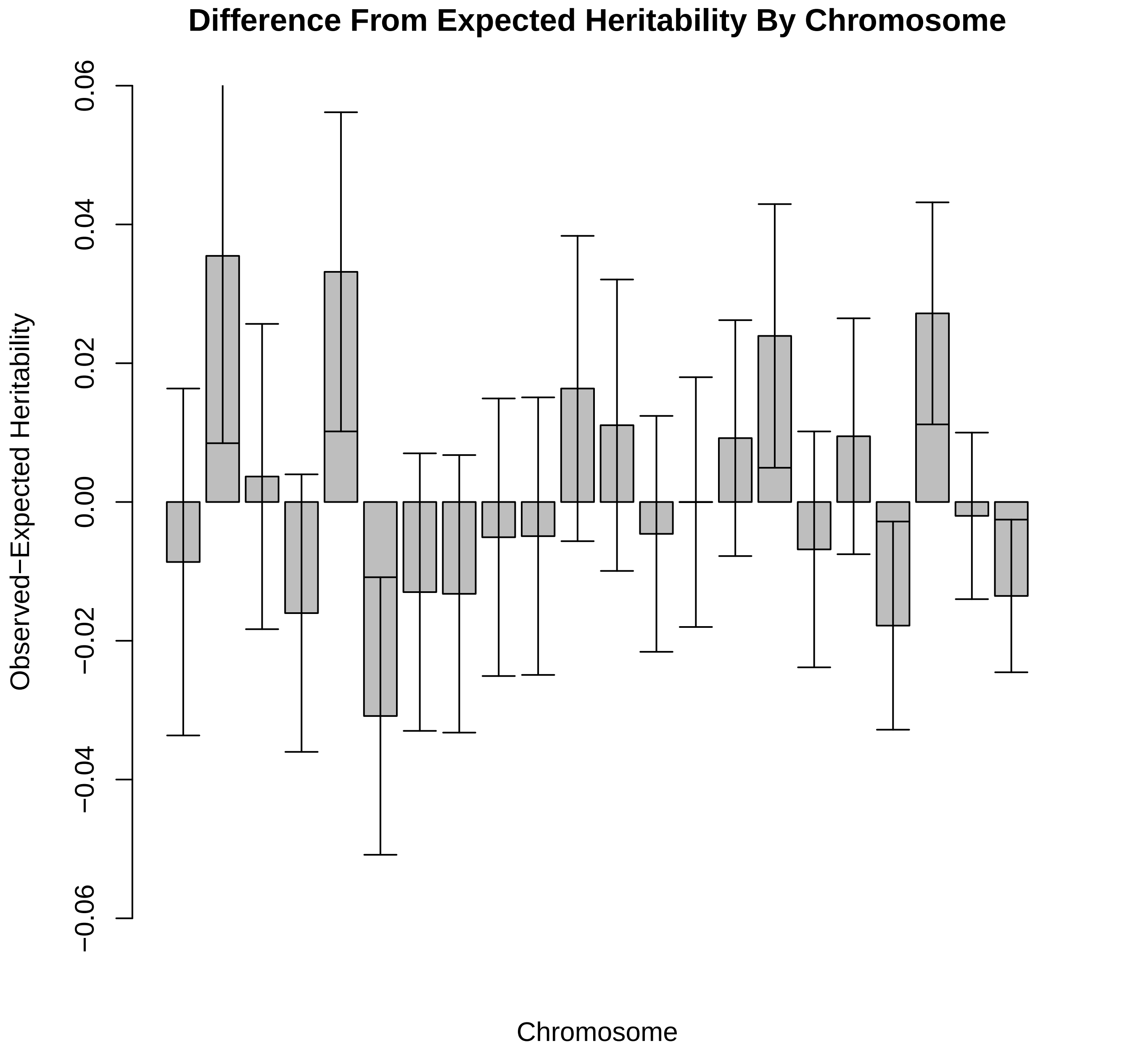

Difference From Expected Heritability By Chromosome
Observed−Expected Heritability
0.06
0.04
0.02
0.00
−0.02
−0.04
−0.06
Chromosome
