# Supplementary material for: Partitioning the Heritability of Tourette Syndrome and Obsessive Compulsive Disorder Reveals Differences in Genetic Architecture
Source: PLoS Genet. 2013 Oct 24;9(10):e1003864. doi: 10.1371/journal.pgen.1003864 (PMC3812053; doi:10.1371/journal.pgen.1003864)
Supplement: Methods S1 — Description of additional methods used in the quality control of samples and SNPs for GCTA analysis. Additionally, a brief description of the identification of eQTL. Finally we provide analytic details of the calculation of heritability on the sibling recurrence risk scale. (DOC) [file pgen.1003864.s011.doc]

**Supplementary Online Methods**

*Additional Quality Control*

We inspected the top eight principal components for outliers or unmatched cases or controls for each diagnosis independently using EIGENSTRAT 3.0 [32]; no poorly matched samples persisted after the data cleaning described in the main text. Since excessive, or differential, relatedness within cases or controls can swamp the IBD estimates and result in incorrect heritability estimates, we examined the pi-hat (i.e., proportion of shared IBD) distributions of all pair-wise relationships (i.e., within cases, within controls, between cases and controls) in an effort to identify any cryptic relatedness in cases and/or controls (Supplementary Figure 2-4). We tested multiple pi-hat cut-offs (ranging from 0.05 to 0.015) applied to the TS and OCD case/control data, to which our results remained robust and stable (Supplementary Table 1).

Finally, we conducted tests of genome-wide identity by state (IBS) stratification using PLINK. This analysis compares average IBS (within and between cases and controls) to average IBS scores from 10,000 permutations of case/control labels. We found no evidence of statistically significant differences in IBS within cases or controls or between cases and controls (all comparison p-values greater than 0.10). Based on these findings we proceeded with analysis of the case-control comparisons using this same QC procedure (Supplementary Table 2).

*Expression Quantitative Trait Locus (eQTL) Identification*

Cerebellar and parietal cortex eQTLs (cis and trans) were generated from 153 individuals of European ancestry obtained from the Stanley Medical Research Institute [38]. Muscle eQTLs were generated from 62 individuals and methods are described in depth elsewhere [39], [40]. Adjustments for batch, as well as known and unknown covariate effects, were performed by ComBat and Surrogate Variable Analysis (SVA) [41], [42]. Expression probes containing common SNPs (MAF > 0.01 in CEU) were removed. Genotyping for the brain samples was performed on the Affymetrix Genome-wide Human SNP Array 5.0 (Affymetrix, Santa Clara, CA, USA) and genotyping for the muscle samples were performed on the Illumina 1M platform. All genotyping was imputed with MaCH to include all HapMap SNPs [44], [45]. Genotyping data for all brain samples used are available on the Stanley Medical Research Institute Online Genomics Database (www.stanleygenomics.org). Gene expression values for parietal cortex (GSE35977), cerebellum (GSE35974), and muscle (GSE40234) were assayed with the Affymetrix Human Gene 1.0 ST Exon Array. Imputed genotype dosage data was analyzed for association with expression phenotypes using PLINK [43]. Cis eQTLs were defined as SNPs within 4 Mb of the expression probe site and otherwise defined as trans eQTLs. The corrected significance for qualification as an eQTL (eQTL p-value) was set at an inclusive threshold of 0.001 in all tissues.

*Calculation of heritability on the sibling recurrence risk scale*

The recurrence risk to first-degree relatives (λ_1st-GCTA_) was calculated based on the the heritability determined by GCTA at a given population prevalence and the population prevalence. For comparison purposes, we also include in supplementary table three the recurrence risk to first-degree relatives calculated based on the heritability from the twin/family studies cited in our paper, at each given population prevalence estimate (λ_1st-lit_)*.* The calculation used for this conversion is given here:

T1=(T-0.5*h2*i)/sqrt(1-0.25*h2*h2*i*(i-T))

Where T = quant norm(K,0,1) and K is the give population prevalence; and h2 is the heritability estimate taken from either the GCTA results or the cited literature. The conversion calculation is drawn from the 1972 paper “The use of multiple thresholds in determining the mode of transmission of semi-continuous traits” by Reich, T., James, J. W. & Morris, C. A.
