# Supplementary material for: Partitioning the Heritability of Tourette Syndrome and Obsessive Compulsive Disorder Reveals Differences in Genetic Architecture
Source: PLoS Genet. 2013 Oct 24;9(10):e1003864. doi: 10.1371/journal.pgen.1003864 (PMC3812053; doi:10.1371/journal.pgen.1003864)
Supplement: Table S1 — Effects of differing pi-hat thresholds on Tourette Syndrome and OCD heritability estimates. Pi-hat refers to the proportion of alleles shared IBD and thus represents a relatedness threshold required for each analysis. (DOC) [file pgen.1003864.s012.doc]

**Supplementary Table 1.** Effects of differing pi-hat thresholds on Tourette Syndrome and OCD heritability estimates. Pi-hat refers to the proportion of alleles shared IBD and thus represents a relatedness threshold required for each analysis.

| **Pi-Hat Threshold** | **TS**  **Heritability**  **(SE)** | **Total TS Sample Size** | **OCD Heritability (SE)** | **Total OCD Sample Size** |
| --- | --- | --- | --- | --- |
| 0.05 | 0.58  (0.08) | 4733 | 0.37  (0.07) | 5297 |
| 0.025 | 0.58  (0.09) | 4675 | 0.37  (0.07) | 5297 |
| 0.02 | 0.58  (0.09) | 4559 | 0.38  (0.08) | 5137 |
| 0.018 | 0.59  (0.09) | 4407 | 0.38  (0.08) | 4936 |
| 0.015 | 0.60  (0.11) | 3372 | 0.44  (0.10) | 3557 |
