# Supplementary material for: Partitioning the Heritability of Tourette Syndrome and Obsessive Compulsive Disorder Reveals Differences in Genetic Architecture
Source: PLoS Genet. 2013 Oct 24;9(10):e1003864. doi: 10.1371/journal.pgen.1003864 (PMC3812053; doi:10.1371/journal.pgen.1003864)
Supplement: Table S2 — Control-control analysis with differing QC thresholds. Table showing changes to the control-control heritability estimate based on differing filtering approaches to the data. Numbers in each cell represent the number of SNPs filtered based on each threshold. MAF = minor allele frequency. Diff SNP Missing = genotypic differential missingness rate. HWD = SNPs with significant deviation (p<0.05) from Hardy Weinberg Equilibrium. SNP Call Rate = Genotyping call rate per sample. Platform Effect SNP = SNPs with significant platform effects. Total # SNPs = Total number of SNPs surviving QC and used in heritability analysis. Total # Sample = Total number of subjects surviving QC and used in heritability analysis. Heritability (se) = Heritability point estimate and standard error of the estimate. P-value = likelihood ration test generated p-value for significance of heritability estimate. (DOC) [file pgen.1003864.s013.doc]

**Supplementary Table 2.** Control-control analysis with differing QC thresholds. Table showing changes to the control-control heritability estimate based on differing filtering approaches to the data. Numbers in each cell represent the number of SNPs filtered based on each threshold. MAF = minor allele frequency. Diff SNP Missing = genotypic differential missingness rate. HWD = SNPs with significant deviation (p<0.05) from Hardy Weinberg Equilibrium. SNP Call Rate = Genotyping call rate per sample. Platform Effect SNP = SNPs with significant platform effects. Total # SNPs = Total number of SNPs surviving QC and used in heritability analysis. Total # Sample = Total number of subjects surviving QC and used in heritability analysis. Heritability (se) = Heritability point estimate and standard error of the estimate. P-value = likelihood ration test generated p-value for significance of heritability estimate.

| **MAF** | **Diff SNP Missing** | **HWD** | **SNP Call Rate** | **Platform Effect SNP** | **Total # SNPs** | **Total # Sample** | **Heritability**  **(se)** | **p-value** |
| --- | --- | --- | --- | --- | --- | --- | --- | --- |
| 16,038  (MAF<0.01) | 50,823  (<0.05) | 18,951  (p<0.05) | 98% | NA | 455,848 | 3,636 | 0.20  (0.06) | 0.0002 |
| 16,038  (MAF<0.01) | 50,823  (<0.05) | 18,951  (p<0.05) | 99.9% | NA | 370,196 | 3,302 | 0.12  (0.06) | 0.016 |
| NA | 50,823  (<0.05) | 18,951  (p<0.05) | 99% | 1951  (p<10^-4^) | 392,120 | 3,294 | 0.00001  (0.06) | 0.5 |
