# Supplementary material for: Partitioning the Heritability of Tourette Syndrome and Obsessive Compulsive Disorder Reveals Differences in Genetic Architecture
Source: PLoS Genet. 2013 Oct 24;9(10):e1003864. doi: 10.1371/journal.pgen.1003864 (PMC3812053; doi:10.1371/journal.pgen.1003864)
Supplement: Table S3 — Heritability for Tourette syndrome, obsessive-compulsive disorder, and early onset obsessive-compulsive disorder at a range of reported population prevalence rates. (DOC) [file pgen.1003864.s014.doc]

**Supplementary Table 3.** Heritability for Tourette syndrome, obsessive-compulsive disorder, and early onset obsessive-compulsive disorder at a range of reported disorder risk rates.

| **Disorder Risk Rate**  **(%)** | **Tourette Syndrome** | | **Obsessive-compulsive disorder** | | **Early onset obsessive-compulsive disorder** | |
| --- | --- | --- | --- | --- | --- | --- |
|  | Heritability  (se) | P-value | Heritability  (se) | P-value | Heritability  (se) | P-value |
| 0.1 | 0.39  (0.06) | 6e-12 | N/A | N/A | N/A | N/A |
| 0.5 | 0.52  (0.08) | 6e-12 | N/A | N/A | N/A | N/A |
| 0.8 | 0.58  (0.09) | 6e-12 | N/A | N/A | N/A | N/A |
| 1.0 | 0.61  (0.09) | 6e-12 | 0.30  (0.06) | 2e-07 | 0.34  (0.08) | 1e-05 |
| 1.5 | N/A | N/A | 0.33  (0.06) | 2e-07 | 0.38  (0.09) | 1e-05 |
| 2.0 | N/A | N/A | 0.35  (0.07) | 2e-07 | 0.41  (0.10) | 1e-05 |
| 2.5 | N/A | N/A | 0.37  (0.07) | 2e-07 | 0.43  (0.10) | 1e-05 |
| 3.0 | N/A | N/A | 0.39  (0.08) | 2e-07 | 0.46  (0.11) | 1e-05 |
