# Supplementary material for: Partitioning the Heritability of Tourette Syndrome and Obsessive Compulsive Disorder Reveals Differences in Genetic Architecture
Source: PLoS Genet. 2013 Oct 24;9(10):e1003864. doi: 10.1371/journal.pgen.1003864 (PMC3812053; doi:10.1371/journal.pgen.1003864)
Supplement: Table S4 — Heritability in terms of sibling recurrence risk (λ) for Tourette syndrome, obsessive-compulsive disorder, and early onset obsessive-compulsive disorder at a range of population prevalences. The title λ1st-GCTA refers to the risk to first degree relatives calculated from the given population prevalence and GCTA based heritability estimate. The title λ1st-lit refers to the risk to first degree relatives calculated from the given population prevalence and the heritability estimates from the literature cited in the main text of the paper. (DOC) [file pgen.1003864.s015.doc]

**Supplementary Table 4.** Heritability in terms of sibling recurrence risk (λ) for Tourette syndrome, obsessive-compulsive disorder, and early onset obsessive-compulsive disorder at a range of population disorder risk rates. The title λ_1st-GCTA_ refers to the risk to first degree relatives calculated from the given population disorder risk rate and GCTA based heritability estimate. The title λ_1st-lit_ refers to the risk to first degree relatives calculated from the given population disorder risk rate and the heritability estimates from the literature cited in the main text of the paper.

| **Population Prevalence**  **(%)** | **Tourette Syndrome** | | **Obsessive-compulsive disorder** | | **Early onset obsessive-compulsive disorder** | |
| --- | --- | --- | --- | --- | --- | --- |
|  | λ1st-GCTA (s.e) | λ1st-lit | λ1st-GCTA (s.e) | λ1st-lit | λ1st-GCTA (s.e) | λ1st-lit |
| 0.1 | 6.61 (1.65) | 14.88 | NA | NA | NA | NA |
| 0.5 | 5.97 (1.37) | 7.46 | NA | NA | NA | NA |
| 0.8 | 5.81 (1.30) | 6.11 | NA | NA | NA | NA |
| 1.0 | 5.69 (1.19) | 5.56 | 2.58 (0.44) | 2.37 | 2.88 (0.63) | 6.24 |
| 1.5 | NA | NA | 2.53 (0.38) | 2.17 | 2.86 (0.62) | 5.20 |
| 2.0 | NA | NA | 2.46 (0.39) | 2.04 | 2.81 (0.61) | 4.58 |
| 2.5 | NA | NA | 2.42 (0.35) | 1.95 | 2.73 (0.55) | 4.15 |
| 3.0 | NA | NA | 2.39 (0.37) | 1.87 | 2.73 (0.56) | 3.83 |
