# Supplementary material for: Partitioning the Heritability of Tourette Syndrome and Obsessive Compulsive Disorder Reveals Differences in Genetic Architecture
Source: PLoS Genet. 2013 Oct 24;9(10):e1003864. doi: 10.1371/journal.pgen.1003864 (PMC3812053; doi:10.1371/journal.pgen.1003864)
Supplement: Table S5 — Tourette Syndrome heritability partitioned by chromosome. Heritability estimates given for each chromosome for both directly genotyped and imputed data. P-values calculated with a likelihood ratio test are also included; * indicates p-values significant after Bonferroni correction. (DOC) [file pgen.1003864.s016.doc]

**Supplementary Table 5.** Tourette Syndrome heritability partitioned by chromosome. Heritability estimates given for each chromosome for both directly genotyped and imputed data. P-values calculated with a likelihood ratio test are also included; * indicates p-values significant after Bonferroni correction.

| **Chr** | **Tourette Syndrome**  **GWAS data** | | **Tourette Syndrome**  **Imputed data** | |
| --- | --- | --- | --- | --- |
|  | Heritability  (SE) | P-value | Heritability  (SE) | P-value |
| 1 | 0.05  (*0.03*) | 0.03 | 0.02  (*0.02*) | 0.2 |
| 2 | 0.08  (*0.03*) | 0.002* | 0.07  (*0.03*) | 0.007 |
| 3 | 0.04  (*0.02*) | 0.05 | 0.03  (*0.02*) | 0.1 |
| 4 | 0.01  (*0.02*) | 0.3 | 0.000001  (*0.02*) | 0.5 |
| 5 | 0.06  (*0.02*) | 0.001* | 0.05  (*0.02*) | 0.01 |
| 6 | 0  (*0.02*) | 0.5 | 0.001  (*0.02*) | 0.5 |
| 7 | 0.02  (*0.02*) | 0.2 | 0.02  (*0.02*) | 0.1 |
| 8 | 0.01  (*0.02*) | 0.3 | 0.01  (*0.02*) | 0.3 |
| 9 | 0.02  (*0.02*) | 0.1 | 0.01  (*0.02*) | 0.2 |
| 10 | 0.02  (*0.02*) | 0.2 | 0.005  (*0.01*) | 0.4 |
| 11 | 0.05  (*0.02*) | 0.005 | 0.06  (*0.02*) | 0.001* |
| 12 | 0.04  (*0.02*) | 0.02 | 0.02  (*0.02*) | 0.09 |
| 13 | 0.01  (*0.02*) | 0.3 | 0.01  (*0.02*) | 0.2 |
| 14 | 0.02  (*0.02*) | 0.08 | 0.01  (*0.02*) | 0.2 |
| 15 | 0.03  (*0.02*) | 0.03 | 0.04  (*0.02*) | 0.02 |
| 16 | 0.05  (*0.02*) | 0.001* | 0.05  (*0.02*) | 0.001* |
| 17 | 0.03  (*0.02*) | 0.03 | 0.03  (*0.02*) | 0.04 |
| 18 | 0.02  (*0.02*) | 0.1 | 0.02  (*0.02*) | 0.08 |
| 19 | 0.002  (*0.02*) | 0.5 | 0.01  (*0.01*) | 0.1 |
| 20 | 0.04  (*0.02*) | 0.002* | 0.04  (*0.02*) | 0.005 |
| 21 | 0.01  (*0.01*) | 0.3 | 0.01  (*0.01*) | 0.2 |
| 22 | 0  (*0.01*) | 0.5 | 0.000001  (*0.01*) | 0.5 |
