# Supplementary material for: Partitioning the Heritability of Tourette Syndrome and Obsessive Compulsive Disorder Reveals Differences in Genetic Architecture
Source: PLoS Genet. 2013 Oct 24;9(10):e1003864. doi: 10.1371/journal.pgen.1003864 (PMC3812053; doi:10.1371/journal.pgen.1003864)
Supplement: Table S6 — Obsessive-compulsive disorder heritability partitioned by chromosome. Heritability estimates given for each chromosome for both directly genotyped and imputed data. P-values calculated with a likelihood ratio test are also included; * indicates p-values significant after Bonferroni correction. (DOC) [file pgen.1003864.s017.doc]

**Supplementary Table 6**. Obsessive compulsive disorder heritability partitioned by chromosome. Heritability estimates given for each chromosome for both directly genotyped and imputed data. P-values calculated with a likelihood ratio test are also included; * indicates p-values significant after Bonferroni correction.

| **Chr** | **Obsessive-Compulsive Disorder**  **GWAS data** | | **Obsessive-Compulsive Disorder**  **Imputed data** | |
| --- | --- | --- | --- | --- |
|  | *Heritability*  *(SE)* | *P-value* | *Heritability*  *(SE)* | *P-value* |
| 1 | 0.04  (*0.02*) | 0.02 | 0.05  (*0.02*) | 0.006 |
| 2 | 0.04  (*0.02*) | 0.04 | 0.05  (*0.02*) | 0.01 |
| 3 | 0.04  (*0.02*) | 0.02 | 0.03  (*0.02*) | 0.05 |
| 4 | 0.01  (*0.02*) | 0.4 | 0.000001  (*0.02*) | 0.5 |
| 5 | 0.01  (*0.02*) | 0.3 | 0.01  (*0.02*) | 0.2 |
| 6 | 0  (*0.02*) | 0.5 | 0.000001  (*0.02*) | 0.5 |
| 7 | 0.01  (*0.02*) | 0.4 | 0.000001  (*0.02*) | 0.5 |
| 8 | 0.02  (*0.02*) | 0.1 | 0.03  (*0.02*) | 0.04 |
| 9 | 0.02  (*0.02*) | 0.1 | 0.02  (*0.02*) | 0.1 |
| 10 | 0.04  (*0.02*) | 0.02 | 0.02  (*0.02*) | 0.1 |
| 11 | 0.004 (*0.01*) | 0.4 | 0.003  (*0.01*) | 0.4 |
| 12 | 0.004  (*0.02*) | 0.4 | 0.000001  (*0.02*) | 0.5 |
| 13 | 0.03  (*0.02*) | 0.03 | 0.01  (*0.02*) | 0.2 |
| 14 | 0.03  (*0.01*) | 0.03 | 0.007  (*0.01*) | 0.3 |
| 15 | 0.05  (*0.02*) | 0.0003* | 0.04  (*0.02*) | 0.007 |
| 16 | 0.02 (*0.01*) | 0.07 | 0.02  (*0.01*) | 0.06 |
| 17 | 0.03  (*0.01*) | 0.01 | 0.02  (*0.01*) | 0.06 |
| 18 | 0.004  (*0.01*) | 0.4 | 0.01  (*0.01*) | 0.2 |
| 19 | 0.02  (*0.01*) | 0.1 | 0.01  (*0.01*) | 0.1 |
| 20 | 0  (*0.01*) | 0.5 | 0.000001  (*0.01*) | 0.5 |
| 21 | 0.001  (*0.009*) | 0.4 | 0.008  (*0.01*) | 0.2 |
| 22 | 0.009  (*0.011*) | 0.2 | 0.01  (*0.01*) | 0.2 |
