# Supplementary material for: Partitioning the Heritability of Tourette Syndrome and Obsessive Compulsive Disorder Reveals Differences in Genetic Architecture
Source: PLoS Genet. 2013 Oct 24;9(10):e1003864. doi: 10.1371/journal.pgen.1003864 (PMC3812053; doi:10.1371/journal.pgen.1003864)
Supplement: Table S7 — GWAS estimated heritability partitioned by genic regions. Heritability estimates for TS and OCD partitioned based on genic annotation. “Genic” includes all coding, intronic, 3′UTR and 5′UTR SNPs. Intergenic is defined as not otherwise genic. The number of SNPs (proportion of total SNPs), heritability, and proportion of total heritability is given for TS and OCD. (DOC) [file pgen.1003864.s018.doc]

**Supplementary Table 7.** GWAS estimated heritability partitioned by genic regions. Heritability estimates for TS and OCD partitioned based on genic annotation. “Genic” includes all coding, intronic, 3’UTR and 5’UTR SNPs. Intergenic is defined as not otherwise genic. The number of SNPs (proportion of total SNPs), heritability, and proportion of total heritability is given for TS and OCD.

| *Region of Interest* | **Tourette Syndrome** | | | **Obsessive-Compulsive Disorder** | | |
| --- | --- | --- | --- | --- | --- | --- |
|  | *Number of SNPs*  *(%)* | *Heritability*  *(SE)* | *Proportion of total*  *heritability* | *Number of SNPs*  *(%)* | *Heritability*  *(SE)* | *Proportion of total heritability* |
| Genic | 178,523  (45%) | 0.30  (*0.07*) | 53% | 169,517  (45%) | 0.15  (*0.06*) | 41% |
| Intergenic | 214,865  (55%) | 0.27  (*0.07*) | 47% | 204,330  (55%) | 0.22  (*0.06*) | 59% |
