# Supplementary material for: Partitioning the Heritability of Tourette Syndrome and Obsessive Compulsive Disorder Reveals Differences in Genetic Architecture
Source: PLoS Genet. 2013 Oct 24;9(10):e1003864. doi: 10.1371/journal.pgen.1003864 (PMC3812053; doi:10.1371/journal.pgen.1003864)
Supplement: Table S8 — Partitioning analysis of heritability based on brain eQTL annotations. Partitions include eQTLs identified in cerebellum only, in parietal cortex only, in both parietal cortex and cerebellum, and non-eQTL SNPs. (DOC) [file pgen.1003864.s019.doc]

**Supplementary Table 8.** Partitioning analysis of heritability based on brain eQTL annotations. Partitions include eQTLs identified in cerebellum only, in parietal cortex only, in both parietal cortex and cerebellum, and non-eQTL SNPs.

| Partition | Number of SNPs  (%) | Tourette syndrome | | | Obsessive-compulsive disorder | | |
| --- | --- | --- | --- | --- | --- | --- | --- |
|  |  | Heritability | Proportion of total heritability estimate | p-value | Heritability | Proportion of total heritability estimate | p-value |
|  |  | (se) |  |  | (se) |  |  |
| Parietal Only eQTL | 158,565  (2.1%) | 0.114 | 27% | 0.03 | 0.084 | 26% | 0.05 |
|  |  | (0.05) |  |  | (0.05) |  |  |
| Cerebellum Only eQTL | 221,928  (2.8%) | 0.039 | 9% | 0.3 | 0.063 | 20% | 0.09 |
|  |  | (0.06) |  |  | (0.05) |  |  |
| Parietal and Cerebellum eQTL | 237,127  (3.1%) | 0.043 | 10% | 0.2 | 0.034 | 11% | 0.2 |
|  |  | (0.05) |  |  | (0.04) |  |  |
| Non-eQTL | 7,039,486  (92%) | 0.269 | 64% | 0.02 | 0.133 | 42% | 0.09 |
|  |  | (0.10) |  |  | (0.10) |  |  |
