# Supplementary material for: Partitioning the Heritability of Tourette Syndrome and Obsessive Compulsive Disorder Reveals Differences in Genetic Architecture
Source: PLoS Genet. 2013 Oct 24;9(10):e1003864. doi: 10.1371/journal.pgen.1003864 (PMC3812053; doi:10.1371/journal.pgen.1003864)
Supplement: Table S9 — Partitioning analysis of heritability based on brain and skeletal muscle eQTL annotations. Partitions include eQTLs identified in brain only, in muscle only, in both brain and muscle, and non-eQTL SNPs. (DOC) [file pgen.1003864.s020.doc]

**Supplementary Table 9.** Partitioning analysis of heritability based on brain and skeletal muscle eQTL annotations. Partitions include eQTLs identified in brain only, in muscle only, in both brain and muscle, and non-eQTL SNPs.

| Partition | Number of SNPs | Tourette syndrome | | | Obsessive-compulsive disorder | | |
| --- | --- | --- | --- | --- | --- | --- | --- |
|  |  | Heritability | Proportion of total heritability estimate | p-value | Heritability | Proportion of total heritability estimate | p-value |
|  |  | (se) |  |  | (se) |  |  |
| Brain Only eQTL | 533,181  (7%) | 0.159 | 33% | 0.06 | 0.190 | 59% | 0.009 |
|  |  | (0.10) |  |  | (0.08) |  |  |
| Muscle Only eQTL | 381,595  (5%) | 0.120 | 25% | 0.1 | 0.084 | 25% | 0.2 |
|  |  | (0.10) |  |  | (0.09) |  |  |
| Brain and Muscle eQTL | 175,268  (2.3%) | 0.037 | 8% | 0.3 | 0.000001 | 0% | 0.5 |
|  |  | (0.08) |  |  | (0.06) |  |  |
| Non-eQTL | 6,567,062  (85.7%) | 0.163 | 34% | 0.2 | 0.05 | 16% | 0.3 |
|  |  | (0.16) |  |  | (0.08) |  |  |
