# Supplementary material for: Partitioning the Heritability of Tourette Syndrome and Obsessive Compulsive Disorder Reveals Differences in Genetic Architecture
Source: PLoS Genet. 2013 Oct 24;9(10):e1003864. doi: 10.1371/journal.pgen.1003864 (PMC3812053; doi:10.1371/journal.pgen.1003864)
Supplement: Table S10 — Proportion heritability and correlation with chromosome length for all phenotypes analyzed with GCTA. Table includes data from representative account of GCTA publications with respective reference, phenotype studied, proportion of total twin/family study heritability estimated by GCTA analysis, correlations reported for heritability by chromosome and chromosome length, adjusted correlation reported for heritability by chromosome and chromosome length (upon removal of outliers). (DOC) [file pgen.1003864.s021.doc]

**Supplementary Table 10.** Proportion heritability and correlation with chromosome length for all phenotypes analyzed with GCTA. Table includes data from representative account of GCTA publications with respective reference, phenotype studied, proportion of total twin/family study heritability estimated by GCTA analysis, correlations reported for heritability by chromosome and chromosome length, adjusted correlation reported for heritability by chromosome and chromosome length (upon removal of outliers).

| **Reference** | **Phenotype** | **% Heritability Accounted For By GCTA Analysis** | **R2 Of Heritability And Chromosome Length** | **Adjusted Correlation** |
| --- | --- | --- | --- | --- |
| Yang et al., 2011 | Height | 47 -52 | 0.695 | NA |
| Yang et al., 2011 | Von Willebrand’s Factor | 33 - 100 | 0.021 | NA |
| Yang et al., 2011 | QTi | 28 - 46 | 0.422 | NA |
| Yang et al., 2011 | BMI | 20 - 38 | 0.076 | NA |
| Lee et al., 2011 | Crohn’s Disease | 40 - 48 | NR | NA |
| Lee et al., 2011 | Bipolar Disorder | 58 | NR | NA |
| Lee et al., 2011 | Type 1 Diabetes | 36 - 44 | NR | NA |
| Lee et al., 2012 | Schizophrenia | 32 - 44 | 0.80 | NA |
| Klei et al., 2012 | Autism | 50 - 100 | 0.46 – 0.54 | NA |
| Keller et al., 2012 | Parkinson’s Disease | 53 - 93 | NR | NA |
| Lee et al., 2013 | Alzheimer’s Disease | 31 | 0.024 | 0.25 (ApoE) |
| Lee et al., 2013 | Endometriosis | 52 | 0.59 | NA |
| Lee et al., 2013 | Multiple Sclerosis | 39 - 100 | 0.37 | 0.45 (Chr 6) |
| Davis et al, (current manuscript) | Tourette Syndrome | 80 - 100 | 0.46 | NA |
| Davis et al, (current manuscript) | Obsessive Compulsive Disorder | 57 - 66 | 0.35 | 0.44 (Chr 15) |
